# An Inducible Retroviral Expression System for Tandem Affinity Purification Mass-Spectrometry-Based Proteomics Identifies Mixed Lineage Kinase Domain-like Protein (MLKL) as an Heat Shock Protein 90 (HSP90) Client*[Fn FN2]

**DOI:** 10.1074/mcp.O115.055350

**Published:** 2015-12-29

**Authors:** Johannes W. Bigenzahn, Astrid Fauster, Manuele Rebsamen, Richard K. Kandasamy, Stefania Scorzoni, Gregory I. Vladimer, André C. Müller, Matthias Gstaiger, Johannes Zuber, Keiryn L. Bennett, Giulio Superti-Furga

**Affiliations:** From the ‡CeMM Research Center for Molecular Medicine of the Austrian Academy of Sciences, Vienna, Austria;; §Department of Biology, Institute of Mol. Syst. Biol., ETH Zürich, Zürich, Switzerland;; ¶Research Institute of Molecular Pathology (IMP), Vienna Biocenter (VBC), 1030 Vienna, Austria;; ‖Center for Physiology and Pharmacology, Medical University of Vienna, Vienna, Austria

## Abstract

Tandem affinity purification–mass spectrometry (TAP-MS) is a popular strategy for the identification of protein–protein interactions, characterization of protein complexes, and entire networks. Its employment in cellular settings best fitting the relevant physiology is limited by convenient expression vector systems. We developed an easy-to-handle, inducible, dually selectable retroviral expression vector allowing dose- and time-dependent control of bait proteins bearing the efficient streptavidin-hemagglutinin (SH)-tag at their N- or C termini. Concomitant expression of a reporter fluorophore allows to monitor bait-expressing cells by flow cytometry or microscopy and enables high-throughput phenotypic assays. We used the system to successfully characterize the interactome of the neuroblastoma RAS viral oncogene homolog (NRAS) Gly12Asp (G12D) mutant and exploited the advantage of reporter fluorophore expression by tracking cytokine-independent cell growth using flow cytometry. Moreover, we tested the feasibility of studying cytotoxicity-mediating proteins with the vector system on the cell death-inducing mixed lineage kinase domain-like protein (MLKL) Ser358Asp (S358D) mutant. Interaction proteomics analysis of MLKL Ser358Asp (S358D) identified heat shock protein 90 (HSP90) as a high-confidence interacting protein. Further phenotypic characterization established MLKL as a novel HSP90 client. In summary, this novel inducible expression system enables SH-tag-based interaction studies in the cell line proficient for the respective phenotypic or signaling context and constitutes a valuable tool for experimental approaches requiring inducible or traceable protein expression.

Protein–protein interactions are the basis of most cellular processes and characterizing the complexes associated with a given protein greatly increases understanding of the biological function (1). Tandem affinity purification (TAP)^1^ (2, 3) coupled to mass spectrometry (MS) constitutes a powerful technique for identifying high-confidence interaction partners of tagged bait proteins (4–6). The reduction of nonspecific background binding due to dual-affinity purification has made TAP-MS the method of choice for protein interaction mapping (7–9), and more than 30 different tandem tags have been established so far by alternative combination of affinity handles (10, 11). Specifically, the purification procedure for the recently developed SH-tag (12) shows particularly high bait protein recovery (10). In combination with the flippase–flippase recognition target (Flp-*FRT*) recombination system, SH-based TAP-MS has been successfully applied to the in-depth analysis of human signaling networks (12–15) and virus–host interactions (16). A detailed interlaboratory comparative analysis of highly standardized procedure using HEK293 cells revealed a reproducibility within an individual laboratory of 98% and a reproducibility between two laboratories of more than 80%, suggesting robustness of the method using workhorse cell lines (15).

Charting the interactome of a specific protein in the relevant physiological setting, in context of its functional signaling pathway, requires performing interaction proteomics in different cellular backgrounds. Highly efficient gene delivery to a variety of cell lines, including cell types that are difficult to transfect, can be achieved by viral-vector-mediated gene transfer (17). Temporal and reversible control of bait protein expression can be achieved by using inducible expression systems, further enabling the analysis of proteins with toxic ectopic expression. Tetracycline (Tet)-On systems (18) have proven to be valuable tools for inducible expression of cDNAs or short hairpin RNAs in cell lines and animal models (19, 20).

To date, TAP-MS experiments are based on Flp-In technology or viral-based transgene delivery of bait proteins fused to different affinity tags with a diverse range of expression and bait recovery efficiency (10, 11, 21). While the SH-tag has comparably high bait recovery (10) and strong interlaboratory reproducibility (15), its application has so far been restricted to the limited number of Flp-In system-competent cell lines. To overcome this limitation and widen the reach of SH-based TAP-MS studies, we established and characterized retroviral expression of SH-tagged proteins for interaction proteomics and color tracing (pRSHIC). This novel retroviral, doxycycline-inducible Tet-On vector system is suitable for expression of SH-tagged target proteins in a wide range of cell systems. In addition to enlarging the existing toolbox for TAP-MS-based interaction proteomics, the features and versatility of pRSHIC make it a valuable tool for a broad set of phenotypic analyses. To illustrate the features of pRSHIC, we charted the interactome of the oncogenic NRAS G12D mutant protein (22, 23), as delineating the network properties of such cancer-associated gene variants is crucial to understand their impact on the disease (24). Furthermore, we demonstrated the applicability of pRSHIC to study cytotoxicity-inducing proteins using the MLKL mutant S358D (25). MLKL is the key molecule required for executing necroptosis, a form of programmed necrotic cell death (26–28). Our study identified MLKL to associate with HSP90 and functionally validated MLKL as a novel client protein of HSP90.

## MATERIALS AND METHODS

### 

#### 

##### Cell Lines and Reagents

HEK293T was obtained from ATCC (Manassas, VA) and K-562 and KCL-22 from DSMZ (Braunschweig, Germany). HT-29 was kindly provided by P. Schneider (Lausanne). Cells were cultured in DMEM (Sigma-Aldrich, St. Louis, MO) or RPMI medium (Sigma-Aldrich) supplemented with 10% (v/v) FBS (Gibco, Grand Island, NY) and antibiotics (100 U/ml penicillin and 100 mg/ml streptomycin) (Sigma-Aldrich). Ba/F3 was obtained from DMSZ and grown in RPMI supplemented with 10% (v/v) FBS (Gibco) and 1–3 ng/ml recombinant murine IL-3 (213–13, PeproTech, Rocky Hill, NJ). The reagents used were as follows: doxycycline (D9891, Sigma-Aldrich), necrostatin-1 (N9037, Sigma-Aldrich), necrosulfonamide (480073, Merck Millipore, Billerica, MA), geldanamycin (G-1047, AG Scientific, San Diego, CA), MG132 (C2211, Sigma Aldrich), chloroquine (C6628, Sigma Aldrich), selumetinib (S1008, Selleck Chemicals, Houston, TX), trametinib (S2673, Selleck Chemicals), and ponatinib (S1490, Selleck Chemicals).

##### Antibodies

Antibodies used were HA (SC-805, Santa Cruz, Dallas, TX), HA-7-HRP (H6533, Sigma-Aldrich), MEK1/2 (#9126, Cell Signaling, Danvers, MA), phospho-MEK1/2 (#2338, Cell Signaling), ERK1/2 (M5670, Sigma-Aldrich), phospho- ERK1/2 (#4370, Cell Signaling), STAT5 (610191, BD Biosciences, Franklin Lakes, NJ), phospho-STAT5A/B (05–886R, Merck Millipore), phospho-p70 S6 kinase (#9234, Cell Signaling), p70 S6 kinase (SC-230, Santa Cruz), RIPK3 (#12107, Cell Signaling), HSP90 (610418, BD Transduction Laboratories), actin (AAN01-A, Cytoskeleton, Denver, CO), and tubulin (ab7291, Abcam, Cambridge, UK). The secondary antibodies used were goat anti-mouse HRP (115–035-003, Jackson ImmunoResearch, West Grove, PA), goat anti-rabbit HRP (111–035-003, Jackson ImmunoResearch), and Alexa Fluor 680 goat anti-mouse (A-21057, Molecular probes, Grand Island, NY).

##### Plasmids and Cloning

Inducible retroviral expression vectors are derived from the pQCXIX self-inactivating retroviral vector backbone (pSIN, Clontech). pRSHIC vectors were assembled using standard cloning techniques and final expression constructs contain the following elements: pSIN-TREtight or TRE3G-HA-StrepII-Gateway cassette-IRES-mCherry-PGK-BlastR for N-terminal StrepHA tagging and pSIN-TREtight or TRE3G-Gateway cassette-StrepII-HA-IRES-mCherry-PGK-BlastR for C-terminal StrepHA tagging. Detailed cloning strategies, primers, and vector information are available upon request. NRAS coding sequence was PCR-amplified from K562 cDNA and cloned into the Gateway-compatible pDONR221 entry vector using BP recombination (Invitrogen, Grand Island, NY). The G12D mutant version of NRAS was generated by site-directed mutagenesis using the QuikChange Lightning Site-Directed Mutagenesis Kit (Agilent Technologies, Santa Clara, CA) using the following primers 5′-GTGGTGGTTGGAGCAGATGGTGTTGGGAAAAGC-3′ and 5′-GCTTTTCCCAACACCATCTGCTCCAACCACCAC-3′. Cloning of RIPK3, MLKL, and MLKL S358D has been described elsewhere (48). Following sequence verification, the cDNAs were transferred by Gateway cloning using LR recombination (Invitrogen) into pRSHIC vectors. All vectors are available upon request.

##### Generation of Inducible Cell Lines

Human cell lines were retrovirally transduced using vector pMSCV-rtTA3-IRES-EcoR-PGK-PuroR (pMSCV-RIEP) (29), and murine cell lines were transduced with pMSCV-rtTA3-PGK-PuroR (pMSCV-RP) (29) to generate rtTA3 and ecotropic receptor-coexpressing (RIEP) or rtTA3-expressing (rtTA3) Tet-on competent cell lines, respectively. Briefly, HEK293T cells were transiently transfected with the retroviral packaging plasmids pGAG-POL, pVSV-G, pADVANTAGE, and pMSCV-RIEP or pMSCV-RP. The medium was exchanged 24 h later and replaced with the medium for the respective target cell line. After 48 h the virus-containing supernatant was harvested, filtered (0.45 μm), supplemented with 8 μg/ml protamine sulfate (Sigma-Aldrich) and added to 40–60% confluent target cell lines. Suspension cells were subjected to spinfection (2000 *rpm*, 45 min, room temperature). 24 h after infection the medium was exchanged and replaced with fresh medium. Another 24 h later, the medium was supplemented with 1–2 μg/ml puromycin (Sigma-Aldrich) to select for infected cells. Following puromycin selection, RIEP- or rtTA3-expressing cell lines were similarly transduced with retrovirus produced in HEK293T cells using the respective target gene-encoding pRSHIC vector, and pGAG-POL, pADVANTAGE, and pEcoEnv. Infected cells were selected by addition of 15–25 μg/ml blasticidin (InvivoGen). Target gene expression was induced by addition of 1–2 μg/ml doxycycline.

##### Immunoblotting

Cells were lysed using Nonidet-40 lysis buffer (50 mm HEPES (pH 7.4), 250 mm NaCl, 5 mm EDTA, 1% Nonidet P-40, 10 mm NaF, 1 mm Na_3_VO_4_, one tablet of EDTA-free protease inhibitor (Roche, Indianapolis, IN, USA) per 50 ml) or IP lysis buffer (50 mm Tris-HCl (pH 7.5), 150 mm NaCl, 5 mm EDTA, 1% Nonidet P-40, 50 mm NaF, 1 mm Na_3_VO_4_, 1 mm PMSF, 5 μg/ml TPCK and protease inhibitor mixture) for 10 min on ice. Lysates were cleared by centrifugation (13000 *rpm*, 10 min, 4 °C). The proteins were quantified with BCA (Pierce, Grand Island, NY) or Bradford assay using γ-globin as a standard (Bio-Rad, Hercules, CA). Cell lysates were resolved by SDS-PAGE and transferred to nitrocellulose membranes Protran BA 85 (GE Healthcare, Little Chalfont, UK). The membranes were immunoblotted with the indicated antibodies. Bound antibodies were visualized with horseradish peroxidase-conjugated secondary antibodies using the ECL Western blotting system (Thermo Scientific, Waltham, MA) or Odyssey Infrared Imager (LI-COR, Lincoln, NE).

##### Immunoprecipitation

Cells were washed in PBS and lysed in ice-cold HENG buffer (50 mm HEPES-KOH (pH 7.9), 150 mm NaCl, 20 mm Na_2_MoO_4_, 2 mm EDTA, 5% glycerol, 0.5% Triton X-100, one tablet of EDTA-free protease inhibitor (Roche) per 50 ml, 20 mm NaF, and 0.4 mm Na_3_VO_4_) for 10 min on ice. Lysates were cleared by centrifugation (13000 *rpm*, 10 min, 4 °C), quantified with BCA (Pierce), and precleared (30 min, 4 °C) on Sepharose6 beads (Sigma-Aldrich). Subsequently, lysates were incubated (3 h, 4 °C) with monoclonal anti-HA agarose antibody (Sigma-Aldrich). Beads were recovered by centrifugation and washed three times with lysis buffer before analysis by SDS-PAGE and immunoblotting.

##### Affinity Purifications and Sample Preparation for Liquid Chromatography Mass Spectrometry

Tandem affinity purifications were performed as previously described (15, 61). Affinity purifications were performed as biological replicates and cell lines expressing SH-tagged GFP were used as negative controls. In brief, cell lines were incubated with 1–2 μg/ml doxycycline for 7–24 h to induce expression of SH-tagged bait proteins. Whole cell extracts were prepared in 50 mm HEPES (pH 8.0), 150 mm NaCl, 5 mm EDTA, 0.5% Nonidet P-40, 50 mm NaF, 1 mm Na_3_VO_4_, 1 mm PMSF, and protease inhibitor mixture. Cell lysates were cleared by centrifugation (13000 *rpm*, 20 min, 4 °C). Proteins were quantitated by Bradford assay using γ-globin as standard (Bio-Rad). 50 mg total lysate were incubated with StrepTactin Sepharose beads (IBA, Göttingen, Germany). Tagged proteins were eluted with d-biotin (Alfa-Aesar, Ward Hill, MA) followed by a second purification step using HA-agarose beads (Sigma-Aldrich). Protein complexes were eluted with 100 mm formic acid and immediately neutralized with triethylammonium bicarbonate buffer (Sigma-Aldrich). Samples were digested with trypsin (Promega, Fitchburg, WI), and the resultant peptides desalted and concentrated with customized reversed-phase tips (62). The volume of the eluted samples was reduced to ∼2 μl in a vacuum centrifuge and reconstituted with 5% formic acid.

##### Reversed-Phase Liquid Chromatography Mass Spectrometry

Mass spectrometry was performed on a hybrid linear trap quadrupole Orbitrap Velos mass spectrometer (ThermoFisher Scientific, Waltham, MA) using the Xcalibur version 2.1.0 coupled to an Agilent 1200 HPLC nanoflow system (dual pump system with one precolumn and one analytical column) (Agilent) via a nanoelectrospray ion source using liquid junction (Proxeon, Odense, Denmark). Solvents for liquid chromatography mass spectrometry separation of the digested samples were as follows: solvent A consisted of 0.4% formic acid in water and solvent B consisted of 0.4% formic acid in 70% methanol and 20% isopropanol. From a thermostatic microautosampler, 8 μl of the tryptic peptide mixture were automatically loaded onto a trap column (Zorbax 300SB-C18 5 μm, 5 × 0.3 mm, Agilent) with a binary pump at a flow rate of 45 μl/min. 0.1% TFA was used for loading and washing the precolumn. After washing, the peptides were eluted by back-flushing onto a 16 cm fused silica analytical column with an inner diameter of 50 μm packed with C18 reversed phase material (ReproSil-Pur 120 C18-AQ, 3 μm, Dr. Maisch, Ammerbuch-Entringen, Germany). The peptides were eluted from the analytical column with a 27 min gradient ranging from 3 to 30% solvent B, followed by a 25 min gradient from 30 to 70% solvent B, and, finally, a 7 min gradient from 70 to 100% solvent B at a constant flow rate of 100 nl/min. The analyses were performed in a data-dependent acquisition mode using a top 15 collision-induced dissociation method. Dynamic exclusion for selected ions was 60 s. A single lock mass at *m/z* 445.120024 was employed (63). The maximal ion accumulation time for MS in the Orbitrap and MS^2^ in the linear trap was 500 and 50 ms, respectively. Automatic gain control was used to prevent overfilling of the ion traps. For MS and MS^2^, automatic gain control was set to 10^6^ and 5,000 ions, respectively. Peptides were detected in MS mode at a resolution of 60,000 (at *m/z* 400). The threshold for switching from MS to MS^2^ was 2,000 counts. All samples were analyzed as technical, back-to-back replicates.

##### Data Analysis

The acquired raw MS data files were processed with msconvert (ProteoWizard Library v2.1.2708) and converted into Mascot generic format (mgf) files. The resultant peak lists were searched against either the human or mouse SwissProt database v2014.03_20140331 (40,055 and 24,830 sequences, respectively, including isoforms obtained from varsplic.pl (64) and appended with known contaminants) with the search engines Mascot (v2.3.02, MatrixScience, London, UK) and Phenyx (v2.5.14, GeneBio, Geneva, Switzerland) (65). Submission to the search engines was via a Perl script that performs an initial search with relatively broad mass tolerances (Mascot only) on both the precursor and fragment ions (±10 ppm and ±0.6 Da, respectively). High-confidence peptide identifications were used to recalibrate all precursor and fragment ion masses prior to a second search with narrower mass tolerances (± 4 ppm and ±0.3 Da, respectively). One missed tryptic cleavage site was allowed. Carbamidomethyl cysteine and oxidized methionine were set as fixed and variable modifications, respectively. To validate the proteins, Mascot and Phenyx output files were processed by internally developed parsers. Proteins with ≤2 unique peptides above a score T1 or with a single peptide above a score T2 were selected as unambiguous identifications. Additional peptides for these validated proteins with score >T3 were also accepted. For Mascot and Phenyx, T1, T2, and T3 peptide scores were equal to 16, 40, 10 and 5.5, 9.5, 3.5, respectively (*p* value <10^−3^). The validated proteins retrieved by the two algorithms were merged and any spectral conflicts discarded and grouped according to shared peptides. By applying the same procedure against a reversed database, a false-positive detection rate of <1 and <0.1% (including the peptides exported with lower scores) was determined for proteins and peptides, respectively. The significance of the interactions from affinity purification-mass spectrometry (AP-MS) experiments was assessed using the SAINT software (51) and the CRAPome database (53). GFP pulldowns were used as the negative control. Commonly known contaminants including trypsin and keratin were removed. Visualization of interaction data was performed using R statistical environment (66). All prey proteins with a SAINT score of >0.95 were identified as high-confidence interactors. Supplemental Tables S1 and S2 give the TAP-LC-MSMS analysis results for NRAS G12D and MLKL S358D, respectively. The mass spectrometry proteomics data have been deposited to the ProteomeXchange Consortium (67) via the PRIDE partner repository with the dataset identifier PXD002855.

##### Cell Viability Assays

Cells were seeded in 96-well plates at the appropriate cell density. For drug sensitivity experiments, cells were incubated with increasing drug concentrations for 72 h. For cell death assays, cells were incubated with the indicated compounds as stated or overnight (14 h). Cell viability was determined using CellTiter Glo Luminescent Cell Viability Assay (Promega) according to the instructions provided by the manufacturer. Luminescence was recorded with a SpectraMax M5Multimode plate reader (Molecular Devices, Sunnyvale, CA). Data were normalized to values of untreated controls.

##### Flow Cytometry

Samples were analyzed on an LSR Fortessa (BD Biosciences), and data analysis was performed using FlowJo software version 7.6.3 (Tree Star Inc., Ashland, OR).

##### Proliferation Competition Assay

To analyze the influence of inducible SH-tagged bait protein expression on cell proliferation and survival, pRSHIC-NRAS G12D (mCherry+) and pRSHIC-GFP (mCherry+/GFP+) transduced Ba/F3 rtTA3 cells were induced with 1 μg/ml doxycycline. After 24 h, cells were mixed in a 1:1 ratio and cultured in the presence of doxycycline with or without IL-3. The percentage of mCherry+ and mCherry+/GFP+ populations was monitored daily by flow cytometry, gating only viable cells (FSC/SCC).

##### Microscopy

Microscopy images were taken at 10× with a Leica DFC310 FX on a Leica DM IL LED microscope (Leica Microsystems, Wetzlar, Germany) or at 20× on an Operetta automated confocal microscope (PerkinElmer, Waltham, MA) and analyzed with ImageJ 1.44p (NIH, open source). The fluorophores used contained no overlapping spectrums and channels were imaged sequentially.

##### Experimental Design and Statistical Rational

Tandem affinity purifications were performed as biological replicates (*n* = 2) and analyzed by LC-MSMS as technical duplicates. Cell viability assay data were normalized to untreated control and are shown as mean value ± s.d. of at least two independent experiments (*n* ≥ 2) performed in triplicates. Flow-cytometry-based proliferation competition assay data are shown as mean value ± s.d. of at least two independent experiments (*n* ≥ 2). Flow cytometry and immunoblot results shown are representative of at least two independent experiments (*n* ≥ 2).

## RESULTS AND DISCUSSION

### 

#### 

##### Generation of a Retroviral Expression System for Inducible, Dose-Dependent, and Reversible Expression of SH-Tagged Bait Proteins

**Fig. 1.**
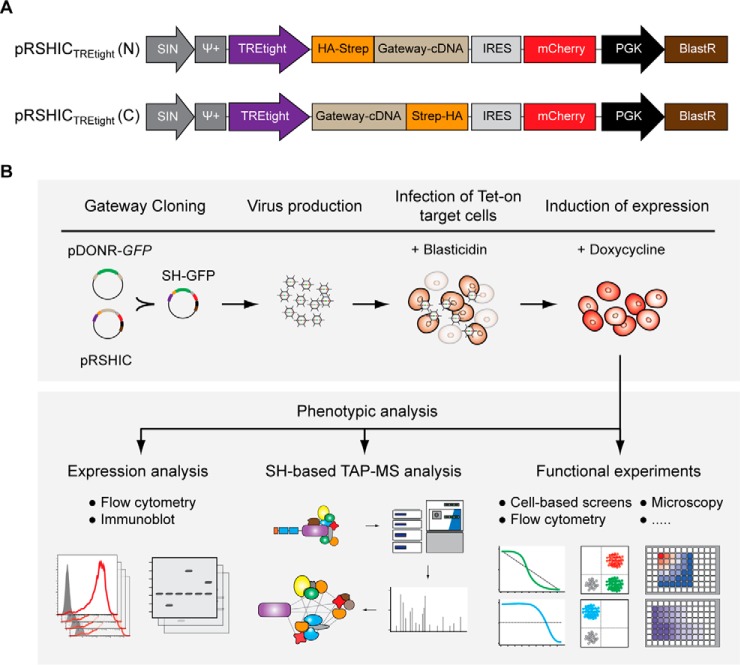
**Main features of pRSHIC and workflow for generation of inducible cell lines.** (*A*) Schematic illustration of inducible TREtight-driven expression vectors with Gateway-cloning cassette fused to N- (upper) or C-terminal (lower) SH-tag. (*B*) Workflow for generation of inducible cell lines amenable to TAP-MS and follow-up experiments.

We assembled an inducible expression system in a self-inactivating retroviral vector containing a tetracycline response element tight (TREtight) promoter (29). For expression of N- or C-terminally TAP-tagged cDNAs, we inserted a gateway-cloning cassette preceded or followed by two streptavidin and one hemagglutinin epitope(s) (12) (Fig. 1*A*). The recombination efficiency of the gateway system allows high-throughput cloning, and thus, the vector is suitable for use with gateway-compatible cDNA and ORF libraries. Furthermore, we linked a fluorescent mCherry marker to the cDNA expression cassette via an internal ribosome entry site (IRES) sequence to enable tracing of bait protein-expressing cell populations by flow cytometry or microscopy. The doxycycline-controlled reverse tet transactivator protein 3 (rtTA3) (30) in combination with different TRE promoters has proven to be effective in inducing transgene expression in a broad range of cell lines and tissues *in vivo* (31). To generate Tet-On proficient cell lines, the respective target cells are first stably transduced with rtTA3 or a combination of rtTA3 and the ecotropic receptor (RIEP), the latter also providing enhanced biosafety (32). Cell lines with inducible bait protein expression are then established by retroviral transduction of rtTA3 transgene-harboring target cells with the respective pRSHIC constructs (Fig. 1*B*). Transduced cells are selected using blasticidin, and transgene expression in the target cell lines can be assessed by flow cytometry or immunoblotting prior to TAP-MS and follow-up experiments.

**Fig. 2.**
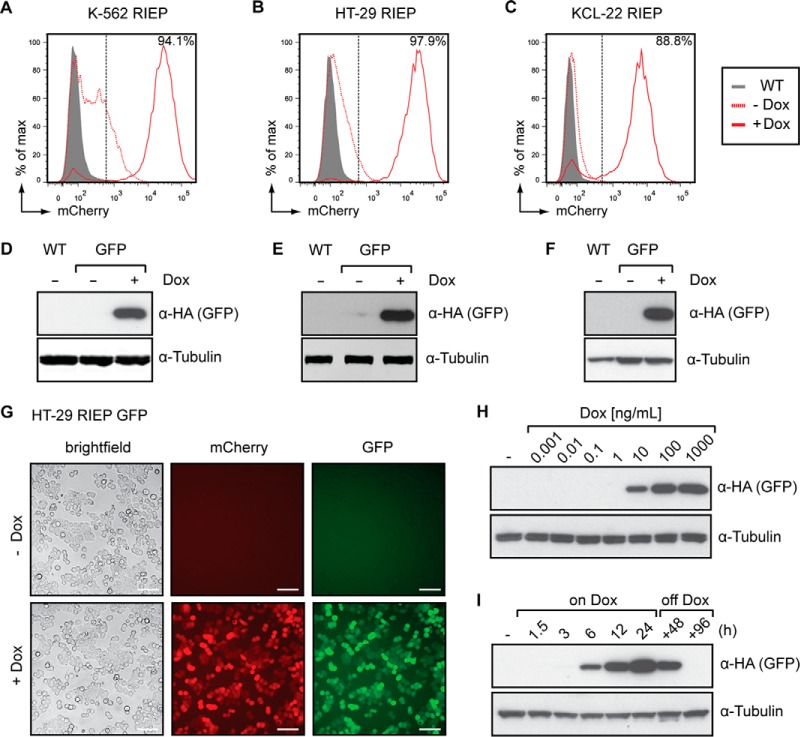
**pRSHIC allows inducible, dose-dependent, and reversible expression of SH-tagged bait proteins.** (*A–F*) Flow cytometry and immunoblot analysis of K-562 RIEP (*A, D*), HT-29 RIEP (*B, E*) and KCL-22 RIEP (*C, F*) GFP cells, untreated or treated with 1–2 μg/ml doxycycline for 24 h. Immunoblots were probed with the indicated antibodies. Wild-type (WT) cells act as a baseline control. (*G*) Microscopy (20×; brightfield, fluorescence) of HT-29 RIEP GFP cells induced or not for 24 h with 2 μg/ml doxycycline (scale bar: 100 μm). (*H*) K-562 RIEP GFP cells were treated with increasing concentrations of doxycycline for 24 h. Cells were lysed and immunoblotted as indicated. (*I*) K-562 RIEP GFP cells were induced with 1 μg/ml and doxycycline subsequently withdrawn for the indicated time span. Cells were lysed and immunoblotted with the indicated antibodies. Results are representative of two independent experiments (*n* = 2).

To characterize the properties of this novel expression system, we transduced human leukemia K-562 RIEP, KCL-22 RIEP and colorectal adenocarcinoma HT-29 RIEP cells with a vector construct encoding SH-tagged green fluorescent protein (GFP). Following selection using blasticidin, the cells were cultured in the presence of doxycycline for 24 h to induce GFP expression. In all three cell lines, >85% of the cell population efficiently induced gene expression as determined by the detection of the mCherry reporter using flow cytometry (Figs. 2*A*–2*C*). Target protein expression was confirmed by immunoblotting for SH-tagged GFP (Figs. 2*D*–2*F*). Additionally, we observed strong correlation between GFP and mCherry fluorescence (Fig. 2*G* and Supplemental Figs. 1*A*–1*C*), indicating that flow cytometry-based detection of the mCherry marker provides a reliable surrogate measure for efficient induction of transgene expression. The TREtight promoter exhibits low basal expression while promoting high-level transcription upon induction. Depending on the promoter used, the efficiency of inducible expression by Tet-regulated systems and the basal expression levels can vary between different cell types (31). For bait proteins with elevated basal expression levels in the context of the TREtight promoter, we additionally created a set of vectors harboring a TRE3G promoter (Supplemental Fig. 2*A*), which provides strongly reduced basal expression compared with earlier versions of the TRE promoter (33) (Supplemental Fig. 2*B*). As demonstrated in K-562 RIEP GFP cells, expression of bait proteins can be modulated by the addition of increasing concentrations of doxycycline (Fig. 2*H*). Furthermore, we monitored induction kinetics, indicating that GFP was induced within hours after doxycycline addition and continued to accumulate over 24 h (Fig. 2*I*). Removal of doxycycline led to a decline in GFP levels, illustrating the reversibility of bait expression (Fig. 2*I*). Altogether, these data establish pRSHIC as a versatile inducible vector system that enables scaling and reversible expression of SH-tagged bait proteins in multiple mammalian cell types.

##### Phenotypic Characterization and Interaction-Proteomic Analysis of NRAS G12D in the Murine Pro B Cell Line Ba/F3

**Fig. 3.**
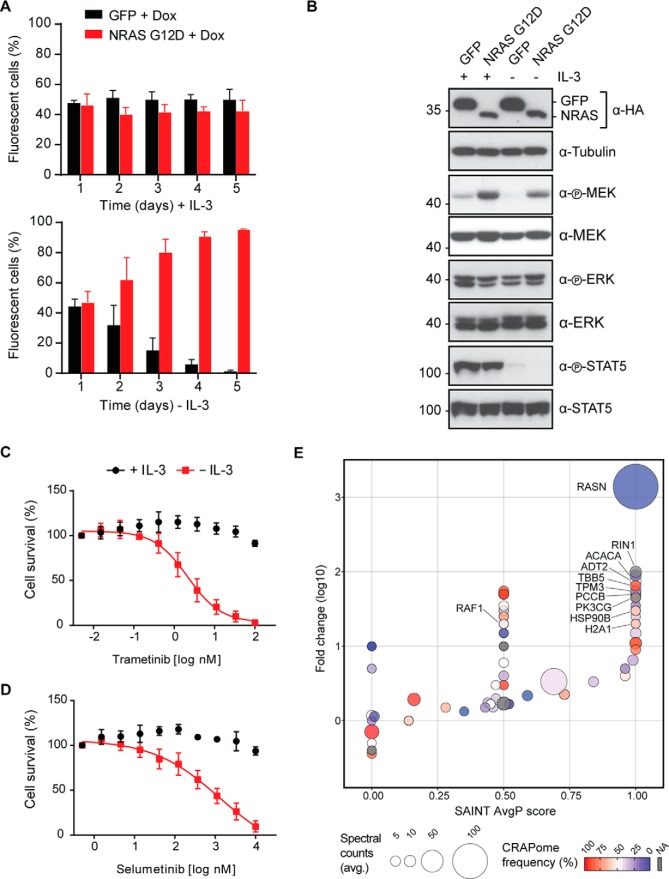
**Phenotypic characterization and interaction-proteomic analysis of NRAS G12D in Ba/F3 cells.** (*A*) Flow cytometry-based proliferation competition assay for Ba/F3 rtTA3 cells expressing NRAS G12D (mCherry+) or GFP (mCherry+/GFP+). After 24 h doxycycline induction cells were mixed at a 1:1 ratio and grown in the presence of 1 μg/ml doxycycline with or without IL-3. The distribution of cell populations was monitored at the indicated time points using flow cytometry. Data represent mean value ± s.d. of at least two independent experiments. (*B*) Ba/F3 rtTA3 GFP and NRAS G12D cells were induced with 1 μg/ml doxycycline in the presence of IL-3 for 48 h. Cells were then washed once, cultured in the presence of 1 μg/ml doxycycline with or without IL-3 for 12h, lysed, and immunoblotted with the indicated antibodies. (*C–D*) Cell viability of Ba/F3 rtTA3 NRAS G12D-expressing cells in the presence or absence of IL-3 upon treatment with trametinib (C) or selumetinib (*D*) as indicated. Data represent mean value ± s.d. of at least two independent experiments performed in triplicates and normalized to untreated control. (*E*) Scatter plot summarizing the SAINT-based significance and CRAPome frequency analysis of NRAS G12D TAP-LC-MSMS experiments. Ba/F3 rtTA3 NRAS cells were grown in presence of IL-3 and induced for 24 h with 1 μg/ml doxycycline. Data shown are based on two independent experiments (*n* = 2), each analyzed as technical duplicates and using Ba/F3 rtTA3 GFP-expressing cells as negative control.

Cancer genome sequencing projects continue to reveal novel gene mutations and fusions (23). Understanding the molecular function of these genetic alterations requires characterization of their phenotypic impact on transformation and specific influence on protein–protein interactions (34, 35). We therefore chose to exemplify utility of pRSHIC through phenotypic analysis of the oncogenic G12D mutant of NRAS, a member of the rat sarcoma (RAS) family (H-, K-, and NRAS) of guanosine triphosphate (GTP)-binding proteins and frequently mutated in hematological malignancies (22). We demonstrated the growth-promoting effects and delineated the interactome of NRAS G12D in the murine bone-marrow-derived pro-B cell line Ba/F3. This cell line requires interleukin (IL)-3 for survival and proliferation and thus constitutes a convenient tool for studying oncogene-induced growth factor independence (36). We generated Tet-On competent Ba/F3 cells inducibly expressing N-terminal SH-tagged NRAS G12D or a GFP control (Supplemental Figs. 3*A* and 3*B*). To examine NRAS G12D-mediated growth factor independence, we performed flow cytometry-based proliferation-competition assays. While both cell populations showed equal growth in the presence of IL-3, NRAS G12D-expressing cells rapidly out-competed GFP-expressing control cells upon IL-3 withdrawal (Fig. 3*A*). Cytokine removal led to loss of signal transducer and activator of transcription 5 (STAT5) phosphorylation in both cell lines; however, NRAS G12D cells maintained elevated mitogen-activated protein kinase (MEK) 1/2 phosphorylation and hence activation of the mitogen-activated protein kinase pathway (Fig. 3*B*). Consequently, NRAS G12D-expressing cells showed marked sensitivity to the MEK 1/2 inhibitors trametinib (GSK1120212) (Fig. 3*C*) and selumetinib (AZD6244) (Fig. 3*D*) in the absence of IL-3, as increasing drug concentrations reduced mitogen-activated protein kinase pathway activation and ribosomal protein S6 kinase 1 (S6K1) phosphorylation (Supplemental Fig. 3*C*). In order to map the interactome of NRAS G12D, we induced bait protein expression for 24 h with doxycycline in the presence of IL-3 and performed TAP coupled to one-dimensional gel-free liquid chromatography tandem mass spectrometry (TAP-LC-MSMS). Significance analysis of interactome (SAINT) analysis using GFP purifications as a control for nonspecific protein interactions identified Ras and Rab interactor 1 (RIN1) among the high-confidence interacting proteins of NRAS G12D (Fig. 3*E* and Supplemental Table 1). Indeed, RIN1 has been described as associating with harvey rat sarcoma viral oncogene homolog (HRAS) and to preferentially bind active, GTP-loaded RAS (37). RIN1 competes with the RAF proto-oncogene serine/threonine-protein kinase (RAF1) for RAS binding (38). Furthermore, we identified phosphatidylinositol 4,5-bisphosphate 3-kinase catalytic subunit gamma isoform (p110γ; PK3CG) of the phosphoinositide-3-kinase (PI3K) complex as a significant interactor. Binding of active RAS isoforms to p110γ leads to activation of the PI3K-pathway (39, 40) and the interaction with p110α (PK3CA) is important for mutant RAS-induced cancer formation and maintenance *in vivo* (41, 42). In summary, by recapitulating the interaction partners and phenotypic features of the oncogenic NRAS G12D protein, we showed that pRSHIC is an efficient tool to functionally annotate and mechanistically characterize proteins bearing cancer-relevant mutations.

##### Phenotypic Analysis of a Cell Death-Inducing MLKL S358D Mutant Protein

**Fig. 4.**
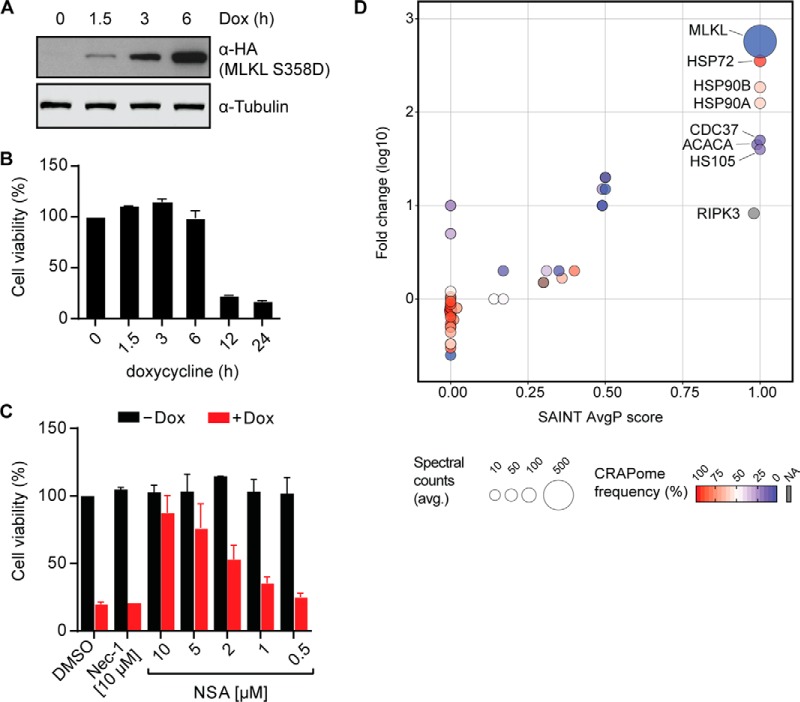
**Phenotypic and TAP-LC-MSMS analysis of the cell death-inducing MLKL S358D mutant.** (*A*) HT-29 RIEP MLKL S358D cells were treated with 2 μg/ml doxycycline for the indicated time. Cells were lysed and immunoblotted with the indicated antibodies. (*B*) Cell viability of HT-29 RIEP MLKL S358D cells induced with 2 μg/ml doxycycline for the indicated time. Data represent mean value ± s.d. of two independent experiments performed as triplicates and normalized to the untreated control. (*C*) Cell viability was examined in HT-29 RIEP MLKL S358D cells untreated or treated overnight with 2 μg/ml doxycycline and the compounds Nec-1 (10 μm) or NSA, as indicated. Data represent the mean value ± s.d. of two independent experiments performed as triplicates and normalized to the untreated control. (*D*) Scatter plot summarizing the SAINT-based significance and CRAPome frequency analysis of MLKL S358D TAP-LC-MSMS experiments. HT-29 RIEP MLKL S358D cells were induced for 7 h with 2 μg/ml doxycycline. Data shown are based on two independent experiments (*n* = 2), each analyzed as technical duplicates with HT-29 RIEP GFP-expressing cells used as the negative control.

The possibility of tightly controlling the timing and extent of protein expression is necessary when investigating proteins that trigger cell death. The pseudokinase MLKL plays a key role in the execution of necroptosis, a form of nonapoptotic programmed cell death relying on the receptor-interacting serine/threonine kinase 1 (RIPK1) and RIPK3 that in recent years has been the subject of very intense research efforts (26–28). Upon activation by RIPK3-mediated phosphorylation, MLKL triggers destabilization and rupture of membranes, resulting in rapid cell death (43–47). We expressed and analyzed a constitutively active MLKL mutant, known to trigger necroptosis (25, 46). We chose to study the RIPK3-phosphorylation mimicking MLKL S358D mutant (48) in the human colorectal adenocarcinoma cell line HT-29, proficient to undergo necroptosis. We observed robust expression of the MLKL S358D mutant in HT-29 RIEP cells within 6 h of doxycycline addition (Fig. 4*A* and Supplemental Fig. 4*A*). As we have shown previously (48), exogenous expression of constitutively active mutant versions of MLKL induces toxicity in these cells. Indeed, MLKL S358D triggered cell death within 12 h after induction as demonstrated by cell viability measurement (Fig. 4*B*) and microscopy (Supplemental Fig. 4*B*). The MLKL inhibitor necrosulfonamide (NSA) (46) inhibited MLKL S358D-induced cell death (48) in a dose-dependent manner (Fig. 4*C*). Conversely, the RIPK1 inhibitor necrostatin-1 (Nec-1) (49) that blocks necroptosis signaling upstream of MLKL, did not confer protection. These data demonstrate that pRSHIC enables expression and, consequently, phenotypic analysis of proteins that promote cell death.

##### TAP-LC-MSMS Analysis Identifies MLKL S358D as an HSP90 Client Protein

**Fig. 5.**
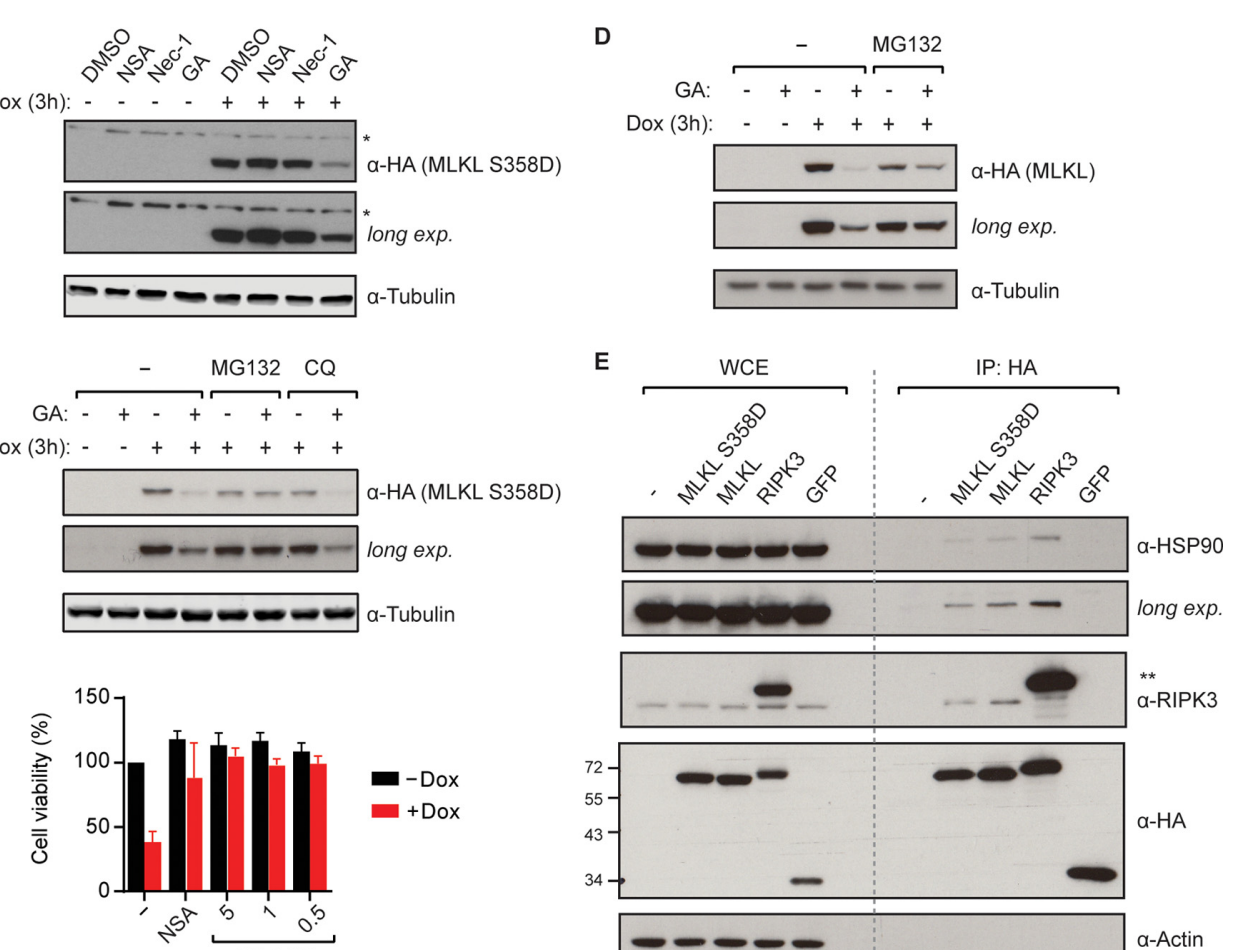
**MLKL is a novel HSP90 client protein.** (*A*) HT-29 RIEP MLKL S358D cells were treated with 2 μg/ml doxycycline and NSA (10 μm), Nec-1 (10 μm) or geldanamycin (GA, 1 μm) for 3 h. Cells were lysed and immunoblotted with the indicated antibodies. Asterisk (*) denotes nonspecific band. Data shown are representative of three independent experiments. (*B*) HT-29 RIEP MLKL S358D cells were pretreated for 1 h with 10 μm MG132 or 10 μm chloroquine (CQ) before induction with 2 μg/ml doxycycline and the addition of 1 μm GA or DMSO. After 3 h of incubation, cells were harvested, lysed, and immunoblotted with the indicated antibodies. Data shown are representative of two independent experiments. (*C*) Cell viability was assessed in HT-29 RIEP MLKL S358D cells induced with 2 μg/ml doxycycline and treated with 10 μm NSA or GA as indicated for 14 h. Data represent mean value ± s.d. of three independent experiments performed as triplicates and normalized to the untreated control. (*D*) HT-29 RIEP MLKL cells were pretreated for 1 h with 10 μm MG132 before induction with 2 μg/ml doxycycline and addition of 1 μm GA or DMSO. After 3 h of incubation, cells were harvested, lysed, and immunoblotted with the indicated antibodies. Data shown are representative of two independent experiments. (*E*) Expression of the indicated bait proteins was induced in HT-29 cells with 2 μg/ml doxycycline for 6 h. Cell lysates were immunoprecipitated and whole cell extracts (WCE) and immunoprecipitates (IP) were analyzed by immunoblotting with the indicated antibodies. Asterisks (**) denote SH-tagged RIPK3. Data shown are representative of two independent experiments.

To identify novel protein interaction partners of MLKL S358D, the cells were induced for 7 h with doxycycline before harvest and TAP-LC-MSMS analysis. The known interactor RIPK3 (47) was significantly enriched in MLKL S358D pulldowns (Fig. 4*D*). Furthermore, heat-shock-related 70 kDa protein 2 (HSP72), HSP90A/B, and the kinase-adaptor cochaperone cell division cycle 37 (CDC37) (50) were identified as high-confidence interactors based on SAINT analysis (51). These heat shock proteins act as molecular chaperones, assisting other proteins to attain and maintain proper folding (52). The comparably high contaminant repository for affinity purification (CRAPome) frequencies (53) assigned to HSP90 and HSP72 likely reflect the large number of client proteins they functionally interact with. Chemical inhibition of HSP90 function leads to client protein destabilization and degradation. Importantly, the HSP90 inhibitor geldanamycin (54) has been shown to block necroptotic cell death (55). This inhibitory effect has been attributed to the destabilizing effect on the two main kinases involved in necroptosis signaling, RIPK1 and RIPK3. Both have been demonstrated to depend on HSP90 (56–58). Our TAP-MS analysis would, however, suggest that the interaction of MLKL with HSP90 may also contribute to this inhibitory effect (Fig. 4*D*). In order to investigate the functional relevance of HSP90 for MLKL S358D, we induced expression in HT-29 RIEP MLKL S358D cells by doxycycline addition for 3 h in the presence of geldanamycin, Nec-1, or NSA. Geldanamycin led to a strong decrease in MLKL S358D protein levels, whereas the other inhibitors had no effect (Fig. 5*A*). To exclude the possibility that geldanamycin interfered with the inducible expression system *per se*, we verified that the mCherry reporter was equally expressed in both control and geldanamycin-treated samples by flow cytometry (Supplemental Fig. 4*C*). The rapid degradation of MLKL S358D upon HSP90 inhibition suggested that this protein constitutes a novel HSP90/CDC37 client. Indeed, the closely related mixed lineage kinase 3 (MLK3) has previously been shown to be stabilized by association with HSP90 and the cochaperone CDC37 (59). The geldanamycin-induced loss of MLKL S358D protein could be prevented by simultaneous treatment with the proteasome inhibitor MG132 (Fig. 5*B*), whereas blocking lysosomal protein degradation using chloroquine had no effect. This data suggested that MLKL S358D was subjected to proteasomal degradation in the absence of HSP90-mediated stabilization, similar to previously described HSP90 client proteins (57). Neither Nec-1 nor ponatinib, recently described to inhibit both RIPK1 and RIPK3 (48, 60), blocked MLKL S358D-induced cell death, indicating that it proceeded independently of these kinases. Yet, the HSP90 inhibitor geldanamycin efficiently blocked MLKL S358D-dependent necroptotic cell death in HT-29 cells (Fig. 5*C*), further corroborating the requirement of HSP90 for MLKL S358D.

Finally, we investigated the requirement of HSP90 function for the MLKL wild-type protein. Similar to the S358D mutant, geldanamycin induced destabilization of the wild-type MLKL protein and this degradation could be blocked by concomitant MG132 treatment (Fig. 5*D*). To confirm the interaction between HSP90 and wild-type MLKL as well as the MLKL S358D mutant, we performed coimmunoprecipitation experiments. MLKL copurified HSP90, similar to the previously described HSP90 client protein RIPK3 (58) (Fig. 5*E*). As demonstrated by the identification and characterization of MLKL as a novel HSP90 client, pRSHIC is an efficient tool to perform phenotypic and TAP-MS analysis of toxicity-promoting proteins.

## CONCLUSIONS

We have established a retroviral-based expression system that expands the repertoire of cell lines amenable to SH-based TAP-MS experiments and thus enables interaction proteomic experiments in the physiologically relevant cellular background. The IRES-linked fluorescent reporter protein allows quick evaluation of bait protein induction by flow cytometry, fluorescence-activated cell sorting of specific cell populations and live tracing of bait-expressing cells to assess phenotypic changes (*i.e.* morphology, surface marker expression, drug resistance). Intracellular localization of the bait proteins can be assessed by probing for the N- or C-terminally fused SH-tag. Moreover, the inducibility of bait expression allows proteins that promote cell death to be studied and opens the opportunity to perform targeted chemical screens in the cell system of choice.

Here, we demonstrated efficiency and applicability of pRSHIC for TAP-MS-based interaction proteomics studies on the oncogenic NRAS G12D mutant protein (22) in murine Ba/F3 cells. Furthermore, we performed interaction proteomics and detailed phenotypic analysis of the cell death-inducing MLKL S358D mutant protein (25) in HT-29 cells, leading to the identification of MLKL as a novel HSP90 client protein.

## Supplementary Material

Supplemental Data

## Footnotes

^1^ The abbreviations used are:

TAP: tandem affinity purification
CDC37: cell division cycle 37
Flp: Flippase
Flp-*FRT*: flippase-flippase recognition target
GFP: green fluorescent protein
GTP: guanosine triphosphate
HSP90: heat shock protein 90
IRES: internal ribosome entry site
MEK: mitogen-activated protein kinase kinase
MLK3: mixed lineage kinase 3
MLKL: mixed lineage kinase domain-like protein
Nec-1: necrostatin-1
NRAS: neuroblastoma RAS viral oncogene homolog
NSA: necrosulfonamide
PK3CG: phosphatidylinositol 4,5-bisphosphate 3-kinase catalytic subunit gamma isoform
RAF1: RAF proto-oncogene serine/threonine-protein kinase
RAS: rat sarcoma
RIN1: Ras and Rab interactor 1
RIPK: receptor-interacting serine/threonine-protein kinase
rtTA3: reverse tet transactivator protein 3
RIEP: rtTA3-IRES-ecotropic receptor-PGK-PuroR
S6K1: ribosomal protein S6 kinase
SH: streptavidin-hemagglutinin-tag
STAT5: signal transducer and activator of transcription 5
TRE: tetracycline-responsive element
pRSHIC: retroviral expression of SH-tagged proteins for interaction proteomics and color-tracing.
