# Supplementary material for: An Inducible Retroviral Expression System for Tandem Affinity Purification Mass-Spectrometry-Based Proteomics Identifies Mixed Lineage Kinase Domain-like Protein (MLKL) as an Heat Shock Protein 90 (HSP90) Client
Source: Mol Cell Proteomics. 2015 Dec 29;15(3):1139–50. doi: 10.1074/mcp.O115.055350 (PMC4813694; doi:10.1074/mcp.O115.055350)
Supplement: Supplemental Data [file supp_15_3_1139__index.html]

An inducible retroviral expression system for tandem affinity purification mass-spectrometry-based proteomics identifies MLKL as an HSP90 client — An Inducible Retroviral Expression System for Tandem Affinity Purification Mass-Spectrometry-Based Proteomics Identifies Mixed Lineage Kinase Domain-like Protein (MLKL) as an Heat Shock Protein 90 (HSP90) Client — pRSHIC Enables Identification of MLKL as HSP90 Client — Supplemental Data 

# An Inducible Retroviral Expression System for Tandem Affinity Purification Mass-Spectrometry-Based Proteomics Identifies Mixed Lineage Kinase Domain-like Protein (MLKL) as an Heat Shock Protein 90 (HSP90) Client

## Supplemental Data

- Supplementary Figures (.pdf, 1.1 MB) - Supplementary Figures
- Supplementary Tables (.pdf, 164 KB) - Supplementary Tables
