# Supplementary material for: An Inducible Retroviral Expression System for Tandem Affinity Purification Mass-Spectrometry-Based Proteomics Identifies Mixed Lineage Kinase Domain-like Protein (MLKL) as an Heat Shock Protein 90 (HSP90) Client
Source: Mol Cell Proteomics. 2015 Dec 29;15(3):1139–50. doi: 10.1074/mcp.O115.055350 (PMC4813694; doi:10.1074/mcp.O115.055350)

Supplementary Figures for "An inducible retroviral expression system for tandem affinity purification mass-spectrometry-based proteomics identifies MLKL as an HSP90 client"

Johannes W. Bigenzahn[1,5], Astrid Fauster[1,5], Manuele Rebsamen[1], Richard K. Kandasamy[1], Stefania Scorzoni[1], Gregory I. Vladimer[1], André C. Müller[1], Matthias Gstaiger[2], Johannes Zuber[3], Keiryn L. Bennett[1], and Giulio Superti-Furga[1,4,*]

[1]CeMM Research Center for Molecular Medicine of the Austrian Academy of Sciences, Vienna, Austria
[2]Department of Biology, Institute of Molecular Systems Biology, ETH Zürich, Zürich, Switzerland
[3]Research Institute of Molecular Pathology (IMP), Vienna Biocenter (VBC), 1030 Vienna, Austria
[4]Center for Physiology and Pharmacology, Medical University of Vienna, Vienna, Austria
[5]These authors contributed equally to this work.

Corresponding author:
Giulio Superti-Furga
CeMM Research Center for Molecular Medicine of the Austrian Academy of Sciences
Lazarettgasse 14, AKH BT25.3
1090 Vienna, Austria
Email: gsuperti@cemm.oeaw.ac.at
Telephone: +43 1 40160 70 001

Supporting Information

Supplementary Figure 1

(**A-C**) FACS analysis of K-562 RIEP (**A**), HT-29 RIEP (**B**) and KCL-22 RIEP (**C**) GFP cells untreated or treated for 24 h with 2 µg/mL doxycycline.

Supplementary Figure 2

(**A**) Schematic illustration of inducible TRE3G-driven pRSHIC expression vectors with Gateway-cloning cassette fused to N- (upper) or C-terminal (lower) SH-tag. (**B**) Flow cytometry analysis of K-562 RIEP transduced with $pRSHIC_{TREtight}$-GFP or $pRSHIC_{TRE3G}$-GFP untreated or treated for 24 h with 1 µg/mL doxycycline. Corresponding wild-type cells are the baseline control.

Supplementary Figure 3

(**A**) Ba/F3 rtTA3 cells inducibly expressing NRAS G12D or GFP were treated with 1 µg/mL doxycycline for 24 h. Cells were lysed and immunoblotted with the indicated antibodies. (**B**) Flow cytometry analysis of Ba/F3 rtTA3 cells expressing NRAS G12D or GFP treated with 1 µg/mL doxycycline for 24 h. (**C**) Ba/F3 rtTA3 NRAS G12D cells were induced with 1 µg/mL doxycycline in the presence of IL-3 for 48 h. Cells were then washed once and cultured in the presence of 1 µg/mL doxycycline and increasing concentrations of trametinib with or without IL-3 for 3 h. DMSO-treated cells were the baseline control. Subsequently cells were lysed and immunoblotted with the indicated antibodies.

Supplementary Figure 4

(**A**) Flow cytometry analysis of HT-29 RIEP MLKL S358D cells induced with 2 µg/mL doxycycline for the indicated times. (**B**) Microscopy (brightfield, 10×) of HT-29 RIEP MLKL S358D cells uninduced or induced with 2 µg/mL doxycycline for 24 h (scale bar: 100 µm). (**C**) Flow cytometry analysis of HT-29 RIEP MLKL S358D cells treated with 2 µg/mL doxycycline and Nec-1 (10 µM), NSA (10µM) or geldanamycin (GA, 1 µM) for 6 h.

Supplementary Figure 1

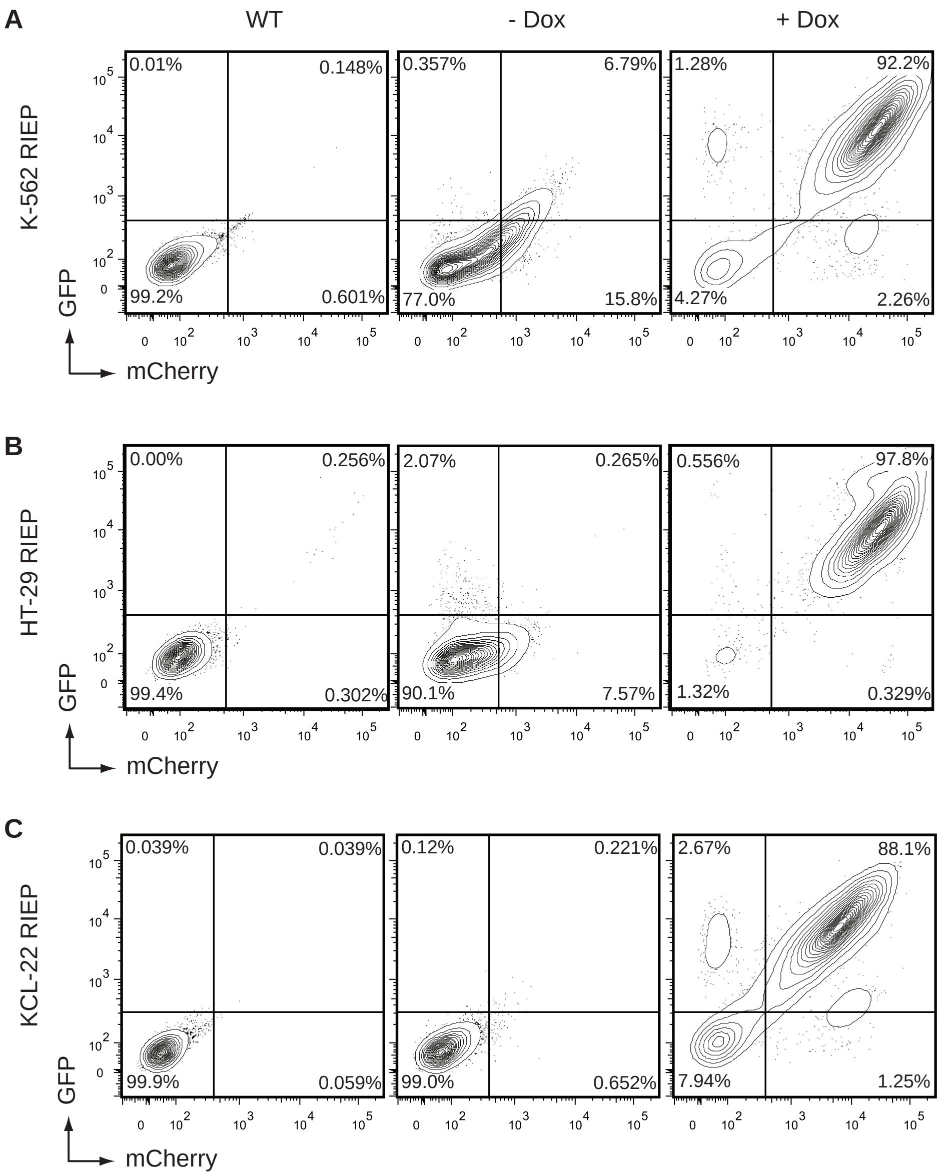

Supplementary Figure 2

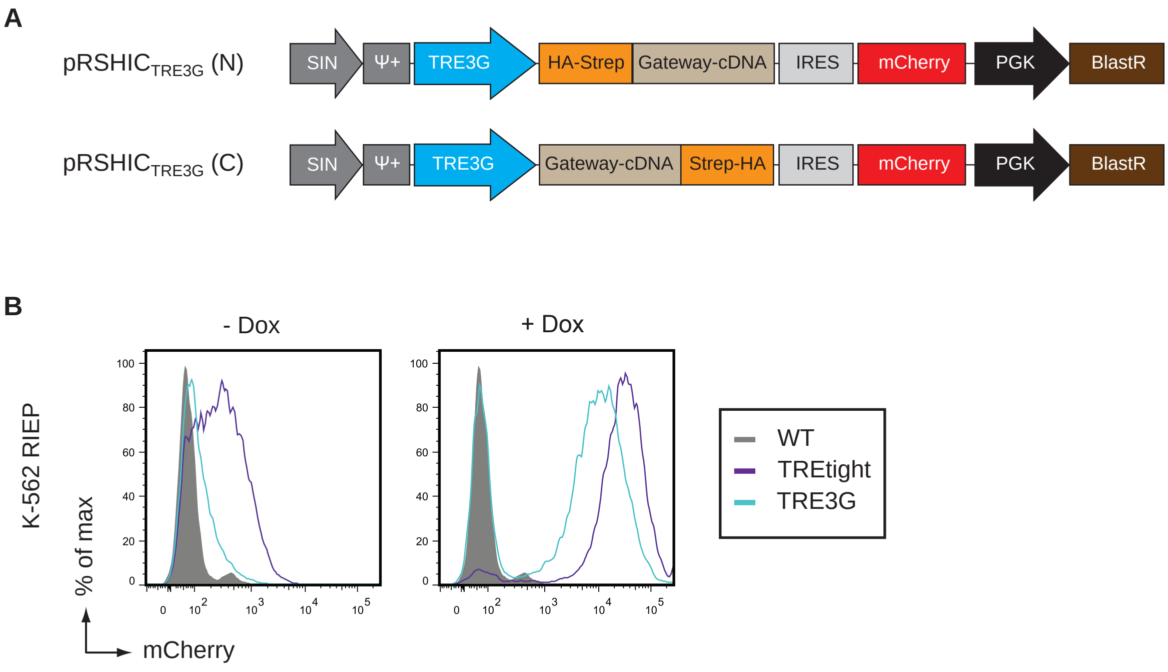

Supplementary Figure 3

A

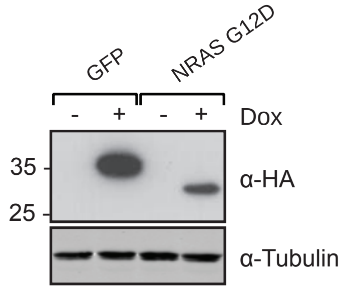

B

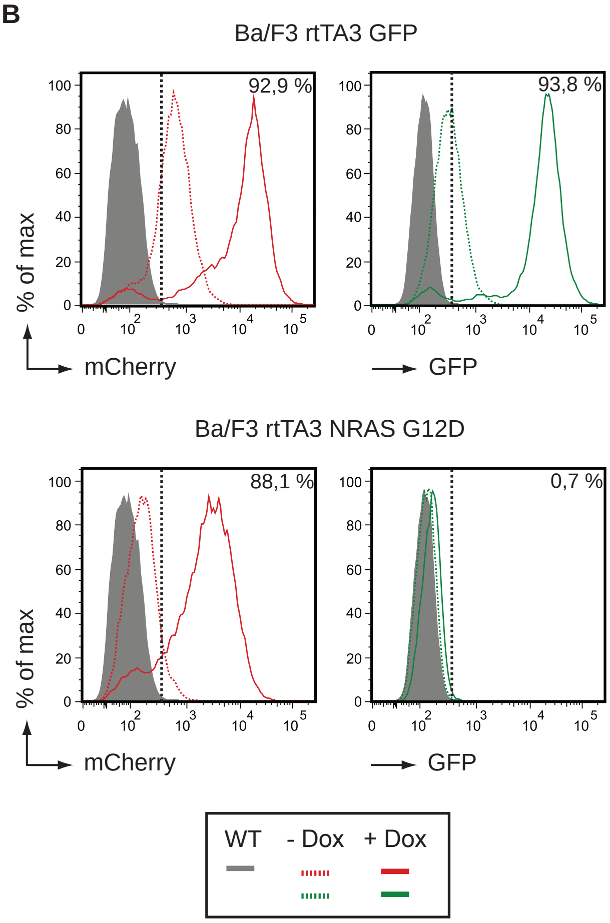

C

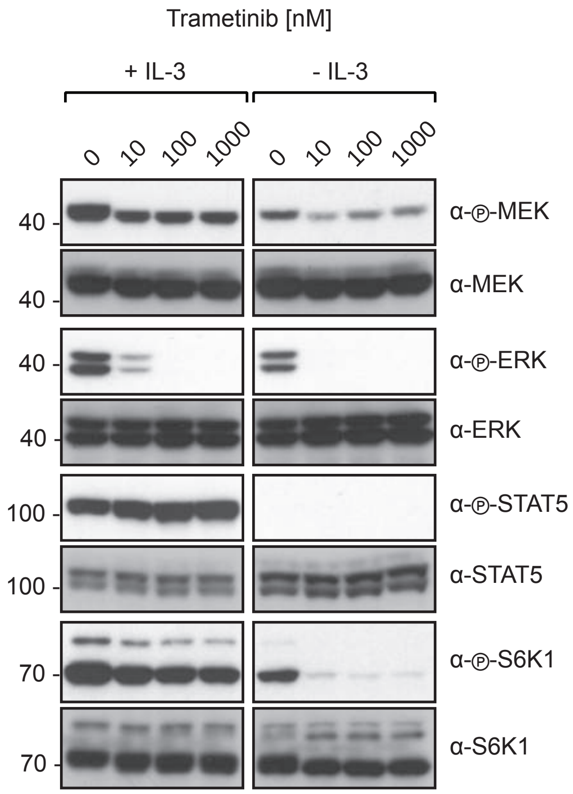

Supplementary Figure 4

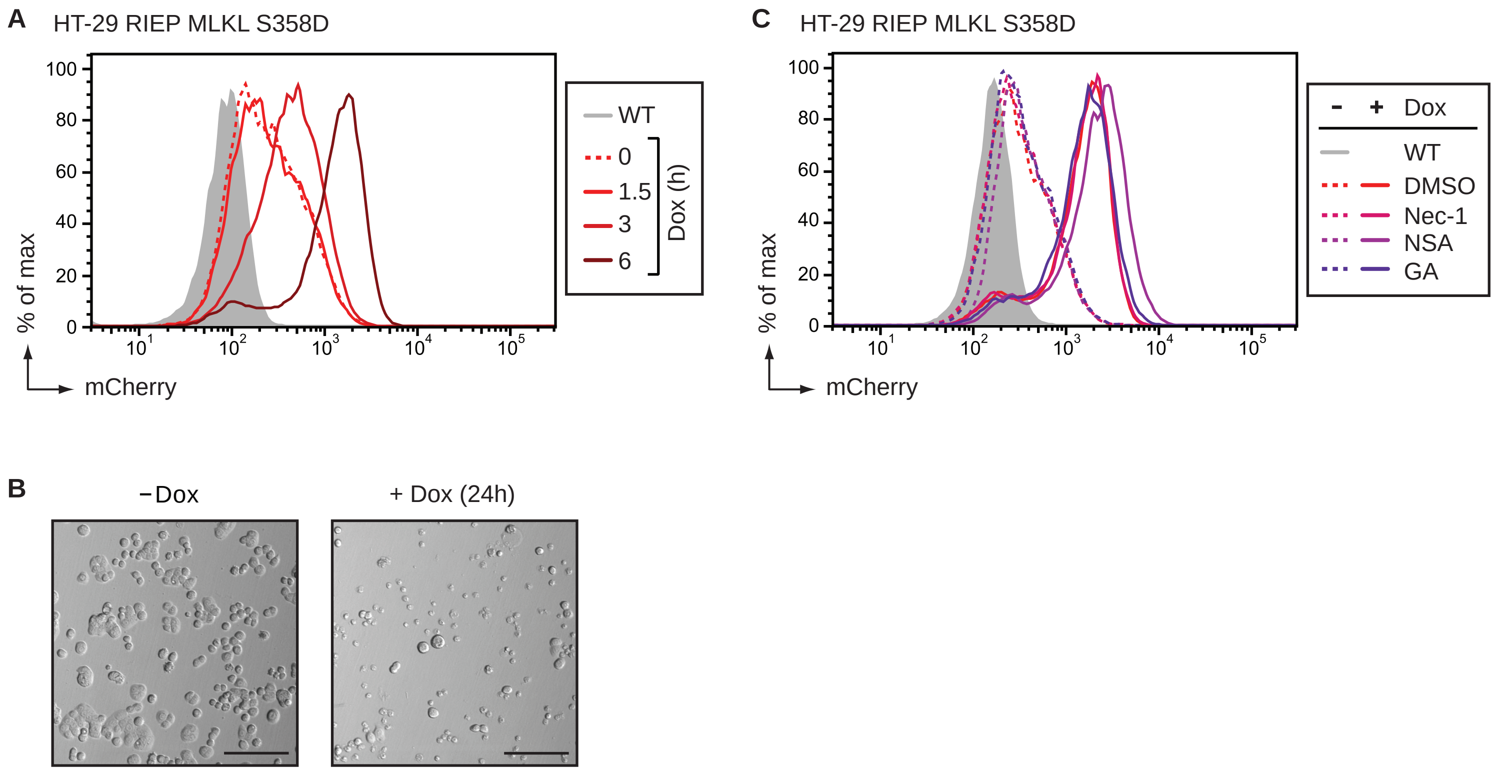

Supplement: Supplemental Data [file 10.1074_O115.055350_mcp.O115.055350-1.pdf]
