# Supplementary material for: An Inducible Retroviral Expression System for Tandem Affinity Purification Mass-Spectrometry-Based Proteomics Identifies Mixed Lineage Kinase Domain-like Protein (MLKL) as an Heat Shock Protein 90 (HSP90) Client
Source: Mol Cell Proteomics. 2015 Dec 29;15(3):1139–50. doi: 10.1074/mcp.O115.055350 (PMC4813694; doi:10.1074/mcp.O115.055350)
Supplement: Supplemental Data [file 10.1074_O115.055350_mcp.O115.055350-2.pdf]

Supplementary Tables for "An inducible retroviral expression system for tandem affinity purification mass-spectrometry-based proteomics identifies MLKL as an HSP90 client"

Johannes W. Bigenzahn[1,5], Astrid Fauster[1,5], Manuele Rebsamen[1], Richard K. Kandasamy[1], Stefania Scorzoni[1], Gregory I. Vladimer[1], André C. Müller[1], Matthias Gstaiger[2], Johannes Zuber[3], Keiryn L. Bennett[1], and Giulio Superti-Furga[1,4,]*

[1]CeMM Research Center for Molecular Medicine of the Austrian Academy of Sciences, Vienna, Austria
[2]Department of Biology, Institute of Molecular Systems Biology, ETH Zürich, Zürich, Switzerland
[3]Research Institute of Molecular Pathology (IMP), Vienna Biocenter (VBC), 1030 Vienna, Austria
[4]Center for Physiology and Pharmacology, Medical University of Vienna, Vienna, Austria
[5]These authors contributed equally to this work.

Corresponding author:
Giulio Superti-Furga
CeMM Research Center for Molecular Medicine of the Austrian Academy of Sciences
Lazarettgasse 14, AKH BT25.3
1090 Vienna, Austria
Email: gsuperti@cemm.oeaw.ac.at
Telephone: +43 1 40160 70 001

Supporting Information

Supplementary Table 1

SAINT and CRAPome analysis of Ba/F3 rtTA3 NRAS G12D TAP-LC-MSMS.

Supplementary Table 2

SAINT and CRAPome analysis of HT-29 RIEP MLKL S358D TAP-LC-MSMS.

Supplementary Table 3

Protein identification of GFP and NRAS G12D TAP-LC-MSMS in Ba/F3 rtTA3 cells.

Supplementary Table 4

Protein identification of GFP and MLKL S358D TAP-LC-MSMS in HT-29 RIEP cells.

Supplementary Table 1
SAINT and CRAPome analysis of Ba/F3 rtTA3 NRAS G12D TAP-LC-MSMS

**Column**	**Description**
Bait	SH-tagged bait protein
PreyID	Identified interactor (UniprotKB ID)
PreyName	Identified interactor (UniprotKB name)
AvgSpec	Average spectral counts of the interactor in two replicate experiments
Spec	Spectral counts of the interactor in two replicate experiments
CtrlCounts	Spectral counts of the interactor in two replicate control experiments
AvgP	SAINT AvgP score
FoldChange	Fold change of the interactor compared to control experiments
CrapomeFreq	Frequency of interactor in CRAPome database

Bait	PreyID	PreyName	AvgSpec	Spec	ctrlCounts	AvgP	FoldChange	CrapomeFreq
RASN (NRAS G12D)	P08556	RASN	138,5	140|137	0|0	1	1385	2,9
RASN (NRAS G12D)	Q921Q7	RIN1	10	11|9	0|0	1	100	NA
RASN (NRAS G12D)	Q5SWU9	ACACA	9	13|5	0|0	1	90	13,1
RASN (NRAS G12D)	P51881	ADT2	7,5	7|8	0|0	1	75	47,2
RASN (NRAS G12D)	P99024	TBB5	6,5	10|3	0|0	1	65	91,5
RASN (NRAS G12D)	P50446	K2C6A	5,5	6|5	0|0	1	55	68,2
RASN (NRAS G12D)	P21107	TPM3	5,5	8|3	0|0	1	55	26,2
RASN (NRAS G12D)	Q8BGZ7	K2C75	5	4|6	0|0	1	50	64,4
RASN (NRAS G12D)	Q99MN9	PCCB	5	6|4	0|0	1	50	16,9
RASN (NRAS G12D)	Q91ZA3	PCCA	4,5	7|2	0|0	1	45	16,6
RASN (NRAS G12D)	P62908	RS3	4,5	7|2	0|0	1	45	58,3
RASN (NRAS G12D)	Q9JHG7	PK3CG	4,5	5|4	0|0	1	45	NA
RASN (NRAS G12D)	Q05920	PYC	3,5	5|2	0|0	1	35	19,8
RASN (NRAS G12D)	P11499	HS90B	3	4|2	0|0	1	30	61,5
RASN (NRAS G12D)	Q03265	ATPA	3	3|3	0|0	1	30	42,9
RASN (NRAS G12D)	Q8VED5	K2C79	3	3|3	0|0	1	30	59,8
RASN (NRAS G12D)	Q791V5	MTCH2	3	4|2	0|0	1	30	3,2
RASN (NRAS G12D)	P61358	RL27	2,5	3|2	0|0	1	25	35,3
RASN (NRAS G12D)	P22752	H2A1	2	2|2	0|0	1	20	60,1
RASN (NRAS G12D)	P62852	RS25	2	2|2	0|0	1	20	44,9
RASN (NRAS G12D)	Q64523	H2A2C	2	2|2	0|0	1	20	60,1
RASN (NRAS G12D)	Q6GSS7	H2A2A	2	2|2	0|0	1	20	60,1
RASN (NRAS G12D)	Q8BFU2	H2A3	2	2|2	0|0	1	20	60,1
RASN (NRAS G12D)	Q8CGP6	H2A1H	2	2|2	0|0	1	20	60,1
RASN (NRAS G12D)	Q8R1M2	H2AJ	2	2|2	0|0	1	20	60,1
RASN (NRAS G12D)	Q8CGP5	H2A1F	2	2|2	0|0	1	20	NA
RASN (NRAS G12D)	Q8CGP7	H2A1K	2	2|2	0|0	1	20	NA
RASN (NRAS G12D)	Q60605	MYL6	7,5	8|7	1|0	1	15	36,7
RASN (NRAS G12D)	P60710	ACTB	11	15|7	2|0	1	11	86
RASN (NRAS G12D)	P63260	ACTG	11	15|7	2|0	1	11	86
RASN (NRAS G12D)	P10126	EF1A1	4,5	5|4	0|1	1	9	82,2
RASN (NRAS G12D)	Q6NXH9	K2C73	6,5	5|8	2|0	0,99	6,5	29,2
RASN (NRAS G12D)	Q9CZX8	RS19	2,5	3|2	1|0	0,96	5	41,4
RASN (NRAS G12D)	Q6IME9	K2C72	2,5	2|3	0|1	0,96	5	22,7
RASN (NRAS G12D)	P16858	G3P	4	3|5	1|1	0,96	4	55,4
RASN (NRAS G12D)	Q01853	TERA	5	7|3	1|2	0,84	3,33	33,5
RASN (NRAS G12D)	P43277	H13	4,5	5|4	2|2	0,73	2,25	67,9
RASN (NRAS G12D)	P15864	H12	4,5	5|4	2|2	0,73	2,25	68,2
RASN (NRAS G12D)	Q8VDD5	MYH9	67	85|49	19|21	0,69	3,35	40,5
RASN (NRAS G12D)	O89023	TPP1	6,5	7|6	2|4	0,59	2,17	0,6
RASN (NRAS G12D)	Q8BMF4	ODP2	2,5	3|2	1|2	0,52	1,67	7,3
RASN (NRAS G12D)	Q99KV1	DJB11	2,5	3|2	2|1	0,52	1,67	7,9
RASN (NRAS G12D)	P68372	TBB4B	5,5	11|0	0|0	0,5	55	89,8
RASN (NRAS G12D)	P68033	ACTC	5	10|0	0|0	0,5	50	83,4
RASN (NRAS G12D)	P68134	ACTS	5	10|0	0|0	0,5	50	83,4
RASN (NRAS G12D)	P68368	TBA4A	5	10|0	0|0	0,5	50	90,1
RASN (NRAS G12D)	Q9JJZ2	TBA8	5	10|0	0|0	0,5	50	89,8
RASN (NRAS G12D)	P62751	RL23A	3,5	6|1	0|0	0,5	35	51,3
RASN (NRAS G12D)	P01837	IGKC	3	6|0	0|0	0,5	30	NA
RASN (NRAS G12D)	P48962	ADT1	3	0|6	0|0	0,5	30	39,7
RASN (NRAS G12D)	Q9Z331	K2C6B	2,5	0|5	0|0	0,5	25	77,6
RASN (NRAS G12D)	Q922U2	K2C5	2,5	0|5	0|0	0,5	25	67,6

RASN (NRAS G12D)	Q99N57	RAF1	2	4|0	0|0	0,5	20	0,9
RASN (NRAS G12D)	P08730	K1C13	2	4|0	0|0	0,5	20	54,8
RASN (NRAS G12D)	P62702	RS4X	2	4|0	0|0	0,5	20	48,4
RASN (NRAS G12D)	Q61753	SERA	1,5	3|0	0|0	0,5	15	29,2
RASN (NRAS G12D)	P11983	TCPA	1,5	2|1	0|0	0,5	15	36,7
RASN (NRAS G12D)	Q01768	NDKB	1,5	3|0	0|0	0,5	15	27,7
RASN (NRAS G12D)	P14115	RL27A	1,5	3|0	0|0	0,5	15	41,4
RASN (NRAS G12D)	P62270	RS18	1,5	3|0	0|0	0,5	15	54,5
RASN (NRAS G12D)	P62900	RL31	1,5	2|1	0|0	0,5	15	36,4
RASN (NRAS G12D)	P35700	PRDX1	1,5	2|1	0|0	0,5	15	54,2
RASN (NRAS G12D)	Q9ESP1	SDF2L	1,5	3|0	0|0	0,5	15	1,2
RASN (NRAS G12D)	Q9DCH4	EIF3F	1	2|0	0|0	0,5	10	17,8
RASN (NRAS G12D)	Q8BH59	CMC1	1	2|0	0|0	0,5	10	6,1
RASN (NRAS G12D)	Q61781	K1C14	1	0|2	0|0	0,5	10	67,1
RASN (NRAS G12D)	P09528	FRIH	1	2|0	0|0	0,5	10	NA
RASN (NRAS G12D)	Q922B2	SYDC	1	2|0	0|0	0,5	10	23,9
RASN (NRAS G12D)	Q61414	K1C15	1	0|2	0|0	0,5	10	54,8
RASN (NRAS G12D)	P38647	GRP75	1	2|0	0|0	0,5	10	69,7
RASN (NRAS G12D)	Q6ZWU9	RS27	1	2|0	0|0	0,5	10	38,2
RASN (NRAS G12D)	P14131	RS16	1	2|0	0|0	0,5	10	42,9
RASN (NRAS G12D)	P62274	RS29	1	2|0	0|0	0,5	10	10,8
RASN (NRAS G12D)	P62281	RS11	1	2|0	0|0	0,5	10	29,4
RASN (NRAS G12D)	P68040	GBLP	1	2|0	0|0	0,5	10	21,9
RASN (NRAS G12D)	Q9QWL7	K1C17	1	0|2	0|0	0,5	10	57,7
RASN (NRAS G12D)	Q9QZD9	EIF3I	1	2|0	0|0	0,5	10	21,6
RASN (NRAS G12D)	Q6ZWY3	RS27L	1	2|0	0|0	0,5	10	33,8
RASN (NRAS G12D)	Q9D5Y0	CG031	1	0|2	0|0	0,5	10	NA
RASN (NRAS G12D)	Q497I4	KRT35	1	2|0	0|0	0,5	10	42
RASN (NRAS G12D)	Q9DCT5	SDF2	1	2|0	0|0	0,5	10	0,6
RASN (NRAS G12D)	Q99KP6	PRP19	1	2|0	0|0	0,5	10	20,4
RASN (NRAS G12D)	P29391	FRIL1	1	2|0	0|0	0,5	10	NA
RASN (NRAS G12D)	Q6IFX2	K1C42	1	0|2	0|0	0,5	10	NA
RASN (NRAS G12D)	P99027	RLA2	3	5|1	1|0	0,5	6	48,4
RASN (NRAS G12D)	O08638	MYH11	4	8|0	0|2	0,5	4	28,6
RASN (NRAS G12D)	P04104	K2C1	3	6|0	2|0	0,5	3	93,3
RASN (NRAS G12D)	P01868	IGHG1	17	28|6	8|12	0,5	1,7	NA
RASN (NRAS G12D)	P01869	IGH1M	17	28|6	8|12	0,5	1,7	NA
RASN (NRAS G12D)	P62830	RL23	1,5	2|1	0|1	0,47	3	51,9
RASN (NRAS G12D)	Q6ZWV7	RL35	1	2|0	1|0	0,47	2	34,4
RASN (NRAS G12D)	P05064	ALDOA	1,5	3|0	2|0	0,46	1,5	34,1
RASN (NRAS G12D)	P62806	H4	2,5	4|1	3|0	0,45	1,67	47,8
RASN (NRAS G12D)	Q3THE2	ML12B	3,5	4|3	2|2	0,44	1,75	30,3
RASN (NRAS G12D)	Q6URW6	MYH14	3	5|1	2|2	0,43	1,5	22,2
RASN (NRAS G12D)	Q9JIY5	HTRA2	2	2|2	1|2	0,35	1,33	0,6
RASN (NRAS G12D)	P43274	H14	3	3|3	2|2	0,28	1,5	68,2
RASN (NRAS G12D)	P05213	TBA1B	14,5	12|17	6|9	0,16	1,93	93,6
RASN (NRAS G12D)	P0CG49	UBB	2	1|3	2|2	0,14	1	58,3
RASN (NRAS G12D)	P0CG50	UBC	2	1|3	2|2	0,14	1	58,3
RASN (NRAS G12D)	P62983	RS27A	2	1|3	2|2	0,14	1	62,1
RASN (NRAS G12D)	P62984	RL40	2	1|3	2|2	0,14	1	58,9
RASN (NRAS G12D)	Q07797	LG3BP	4	4|4	2|5	0,01	1,14	4,7
RASN (NRAS G12D)	P10518	HEM2	1	1|1	0|0	0	10	NA
RASN (NRAS G12D)	P67984	RL22	1	1|1	0|0	0	10	38,8
RASN (NRAS G12D)	P62267	RS23	1	1|1	0|0	0	10	31,2
RASN (NRAS G12D)	Q9CXW4	RL11	1	1|1	0|0	0	10	43,1
RASN (NRAS G12D)	Q9CR62	M2OM	1	1|1	0|0	0	10	7,6
RASN (NRAS G12D)	Q3TCN2	PLBL2	1	1|1	0|0	0	10	0,6
RASN (NRAS G12D)	Q8VBV7	CSN8	1	1|1	0|0	0	10	3,8
RASN (NRAS G12D)	P63276	RS17	0,5	1|0	0|0	0	5	34,7
RASN (NRAS G12D)	P17809	GTR1	0,5	1|0	0|0	0	5	2,9
RASN (NRAS G12D)	P34884	MIF	0,5	0|1	0|0	0	5	0,3
RASN (NRAS G12D)	P34884	MIF	0,5	0|1	0|0	0	5	7,9
RASN (NRAS G12D)	Q9JKB3	YBOX3	0,5	1|0	0|0	0	5	41,7
RASN (NRAS G12D)	P35979	RL12	0,5	1|0	0|0	0	5	40,8
RASN (NRAS G12D)	Q91VR2	ATPG	0,5	1|0	0|0	0	5	9,6
RASN (NRAS G12D)	Q99KQ4	NAMPT	0,5	1|0	0|0	0	5	5,8
RASN (NRAS G12D)	P80318	TCPG	0,5	1|0	0|0	0	5	33,5
RASN (NRAS G12D)	P61982	1433G	0,5	1|0	0|0	0	5	22,4

RASN (NRAS G12D)	P62301	RS13	0,5	1|0	0|0	0	5	35
RASN (NRAS G12D)	Q9JJI8	RL38	0,5	0|1	0|0	0	5	32,1
RASN (NRAS G12D)	P62960	YBOX1	0,5	1|0	0|0	0	5	53,6
RASN (NRAS G12D)	Q8VEK3	HNRPU	0,5	1|0	0|0	0	5	61,8
RASN (NRAS G12D)	Q99MN1	SYK	0,5	1|0	0|0	0	5	12,8
RASN (NRAS G12D)	O54941	SMCE1	0,5	1|0	0|0	0	5	3,8
RASN (NRAS G12D)	Q78IK2	USMG5	0,5	1|0	0|0	0	5	0,6
RASN (NRAS G12D)	Q99JR1	SFXN1	0,5	1|0	0|0	0	5	5
RASN (NRAS G12D)	Q9Z2C8	YBOX2	0,5	1|0	0|0	0	5	40,5
RASN (NRAS G12D)	P52480	KPYM	7	8|6	6|6	0	1,17	52,8
RASN (NRAS G12D)	P61514	RL37A	1	1|1	2|0	0	1	21,3
RASN (NRAS G12D)	P63017	HSP7C	21	23|19	29|30	0	0,71	95,6
RASN (NRAS G12D)	O35658	C1QBP	0,5	1|0	1|1	0	0,5	48,1
RASN (NRAS G12D)	P18524	HVM53	1	1|1	2|3	0	0,4	NA
RASN (NRAS G12D)	P18526	HVM55	1	1|1	2|3	0	0,4	NA
RASN (NRAS G12D)	P20029	GRP78	2,5	5|0	7|7	0	0,36	85,1

Supplementary Table 2
SAINT and CRAPome analysis of HT-29 RIEP MLKL S358D TAP-LC-MSMS

**Column**	**Description**
Bait	SH-tagged bait protein
PreyID	Identified interactor (UniprotKB ID)
PreyName	Identified interactor (UniprotKB name)
AvgSpec	Average spectral counts of the interactor in two replicate experiments
Spec	Spectral counts of the interactor in two replicate experiments
CtrlCounts	Spectral counts of the interactor in two replicate control experiments
AvgP	SAINT AvgP score
FoldChange	Fold change of the interactor compared to control experiments
CrapomeFreq	Frequency of interactor in CRAPome database

Bait	PreyID	PreyName	AvgSpec	Spec	ctrlCounts	AvgP	FoldChange	CrapomeFreq
MLKL (MLKL S358D)	Q8NB16	MLKL	573	537|609	0|2	1	573	0,3
MLKL (MLKL S358D)	P54652	HSP72	35,5	33|38	0|0	1	355	92,7
MLKL (MLKL S358D)	P08238	HS90B	18,5	14|23	0|0	1	185	61,5
MLKL (MLKL S358D)	P07900	HS90A	12,5	10|15	0|0	1	125	60,1
MLKL (MLKL S358D)	Q16543	CDC37	5	5|5	0|0	1	50	11,1
MLKL (MLKL S358D)	Q92598	HS105	4	4|4	0|0	1	40	17,5
MLKL (MLKL S358D)	Q13085	ACACA	4,5	2|7	0|0	0,99	45	13,1
MLKL (MLKL S358D)	Q9Y572	RIPK3	16,5	14|19	0|4	0,98	8,25	NA
MLKL (MLKL S358D)	P08779	K1C16	2	0|4	0|0	0,5	20	64,1
MLKL (MLKL S358D)	Q58FF8	H90B2	2	0|4	0|0	0,5	20	NA
MLKL (MLKL S358D)	Q9H254	SPTN4	2	0|4	0|0	0,5	20	5,2
MLKL (MLKL S358D)	Q8NCM8	DYHC2	1,5	0|3	0|0	0,5	15	0,6
MLKL (MLKL S358D)	P30048	PRDX3	1,5	1|2	0|0	0,49	15	22,7
MLKL (MLKL S358D)	O00294	TULP1	1	0|2	0|0	0,49	10	0,3
MLKL (MLKL S358D)	P08708	RS17	1	0|2	0|0	0,49	10	34,7
MLKL (MLKL S358D)	P0CW22	RS17L	1	0|2	0|0	0,49	10	34,7
MLKL (MLKL S358D)	P62280	RS11	1	0|2	0|0	0,49	10	29,4
MLKL (MLKL S358D)	Q5M9N0	CD158	1	2|0	0|0	0,49	10	0,6
MLKL (MLKL S358D)	P35527	K1C9	2	0|4	2|0	0,4	2	81,9
MLKL (MLKL S358D)	P02533	K1C14	2,5	0|5	0|3	0,36	1,67	67,1
MLKL (MLKL S358D)	Q08380	LG3BP	2	2|2	0|2	0,35	2	4,7
MLKL (MLKL S358D)	P55072	TERA	2	1|3	2|0	0,31	2	33,5
MLKL (MLKL S358D)	P68104	EF1A1	1,5	0|3	1|1	0,3	1,5	82,2
MLKL (MLKL S358D)	Q05639	EF1A2	1,5	0|3	1|1	0,3	1,5	80,2
MLKL (MLKL S358D)	Q5VTE0	EF1A3	1,5	0|3	1|1	0,3	1,5	NA
MLKL (MLKL S358D)	Q9UBS4	DJB11	4	4|4	2|2	0,17	2	7,9
MLKL (MLKL S358D)	P19013	K2C4	1	0|2	0|2	0,17	1	45,5
MLKL (MLKL S358D)	P05387	RLA2	1	0|2	1|1	0,14	1	48,4
MLKL (MLKL S358D)	P02538	K2C6A	2	0|4	1|4	0,02	0,8	68,2
MLKL (MLKL S358D)	P48668	K2C6C	2	0|4	1|4	0,02	0,8	68,2
MLKL (MLKL S358D)	P04259	K2C6B	1,5	0|3	1|4	0,01	0,6	77,6
MLKL (MLKL S358D)	P11142	HSP7C	55	53|57	80|66	0	0,75	95,6
MLKL (MLKL S358D)	P08107	HSP71	28,5	28|29	41|34	0	0,76	95,6
MLKL (MLKL S358D)	P17066	HSP76	25	26|24	32|30	0	0,81	91,3
MLKL (MLKL S358D)	P34931	HS71L	21	19|23	26|26	0	0,81	93,9
MLKL (MLKL S358D)	P11021	GRP78	17,5	17|18	25|26	0	0,69	85,1
MLKL (MLKL S358D)	P68363	TBA1B	15	17|13	20|14	0	0,88	93,6
MLKL (MLKL S358D)	P07437	TBB5	11	8|14	14|7	0	1,05	91,5
MLKL (MLKL S358D)	P62829	RL23	9	11|7	7|8	0	1,2	51,9
MLKL (MLKL S358D)	P68366	TBA4A	8	8|8	15|10	0	0,64	90,1
MLKL (MLKL S358D)	P68371	TBB4B	7,5	4|11	11|5	0	0,94	89,8
MLKL (MLKL S358D)	P13645	K1C10	6,5	0|13	4|22	0	0,5	86,6
MLKL (MLKL S358D)	P0CG47	UBB	5	6|4	16|14	0	0,33	58,3
MLKL (MLKL S358D)	P0CG48	UBC	5	6|4	16|14	0	0,33	58,3
MLKL (MLKL S358D)	P62979	RS27A	5	6|4	16|14	0	0,33	62,1
MLKL (MLKL S358D)	P62987	RL40	5	6|4	16|14	0	0,33	58,9
MLKL (MLKL S358D)	P38646	GRP75	4	4|4	10|8	0	0,44	69,7
MLKL (MLKL S358D)	P04264	K2C1	3,5	0|7	4|9	0	0,54	93,3
MLKL (MLKL S358D)	P35908	K22E	1,5	0|3	1|9	0	0,3	86,9
MLKL (MLKL S358D)	O14773	TPP1	1	1|1	1|1	0	1	0,6
MLKL (MLKL S358D)	P05787	K2C8	1	0|2	2|4	0	0,33	51,9

MLKL (MLKL S358D)	P35030	TRY3	1	1|1	0|0	0	10	16,3
MLKL (MLKL S358D)	P46782	RS5	1	1|1	0|0	0	10	32,7
MLKL (MLKL S358D)	P52597	HNRPF	1	1|1	0|0	0	10	51,3
MLKL (MLKL S358D)	P61247	RS3A	1	1|1	0|0	0	10	41,1
MLKL (MLKL S358D)	P61978	HNRPK	1	1|1	0|0	0	10	64,1
MLKL (MLKL S358D)	P62913	RL11	1	1|1	0|0	0	10	43,1
MLKL (MLKL S358D)	Q06830	PRDX1	1	1|1	0|0	0	10	54,2
MLKL (MLKL S358D)	Q12931	TRAP1	1	1|1	0|0	0	10	15,2
MLKL (MLKL S358D)	Q14257	RCN2	1	1|1	0|0	0	10	27,1
MLKL (MLKL S358D)	O43390	HNRPR	0,5	1|0	0|0	0	5	37,6
MLKL (MLKL S358D)	O43464	HTRA2	0,5	0|1	2|2	0	0,25	0,6
MLKL (MLKL S358D)	O60884	DNJA2	0,5	0|1	0|0	0	5	16,6
MLKL (MLKL S358D)	P05141	ADT2	0,5	0|1	2|0	0	0,5	47,2
MLKL (MLKL S358D)	P05161	ISG15	0,5	0|1	0|0	0	5	1,5
MLKL (MLKL S358D)	P07355	ANXA2	0,5	0|1	0|0	0	5	26,2
MLKL (MLKL S358D)	P12235	ADT1	0,5	0|1	2|0	0	0,5	39,7
MLKL (MLKL S358D)	P12236	ADT3	0,5	0|1	2|0	0	0,5	41,4
MLKL (MLKL S358D)	P13647	K2C5	0,5	0|1	1|0	0	1	67,6
MLKL (MLKL S358D)	P14618	KPYM	0,5	1|0	0|0	0	5	52,8
MLKL (MLKL S358D)	P23396	RS3	0,5	0|1	0|0	0	5	58,3
MLKL (MLKL S358D)	P35232	PHB	0,5	0|1	0|0	0	5	16,9
MLKL (MLKL S358D)	P51784	UBP11	0,5	0|1	0|0	0	5	4,1
MLKL (MLKL S358D)	P61513	RL37A	0,5	0|1	0|0	0	5	21,3
MLKL (MLKL S358D)	P62249	RS16	0,5	0|1	0|0	0	5	42,9
MLKL (MLKL S358D)	P62854	RS26	0,5	0|1	0|0	0	5	29,7
MLKL (MLKL S358D)	P63208	SKP1	0,5	0|1	0|0	0	5	27,7
MLKL (MLKL S358D)	Q9H0C2	ADT4	0,5	0|1	0|0	0	5	34,4

Supplementary Table 3
Protein identification of GFP and NRAS G12D TAP-LC-MSMS in Ba/F3 rtTA3 cells

Column	Description
UniProtID	UniprotKB ID
Entry_name	UniprotKB entry name
Gene_names	UniprotKB entry derived gene names
AvgSeqCov	Average sequence coverage in two technical replicates
AvgSpecCount	Average spectral counts in two technical replicates
AvgPepCount	Average peptide counts in two technical replicates

GFP TAP-LC-MSMS in Ba/F3 rtTA3 cells, 1st biological replicate

UniProtID	Entry_name	Gene_names	AvgSeqCov	AvgSpecCount	AvgPepCount
O08638	MYH11_MOUSE	Myh11	1	2	2
O35658	C1QBP_MOUSE	C1qbp Gc1qbp	5	1,5	1
O89023	TPP1_MOUSE	Tpp1 Cln2	8	4	2,5
P01868	IGHG1_MOUSE	Ighg1 Igh-4	19	12,5	5,5
P01869	IGH1M_MOUSE	Ighg1 Igh-4	15	12,5	5,5
P05213	TBA1B_MOUSE	Tuba1b Tuba2	18	9,5	5,5
P0CG49	UBB_MOUSE	Ubb	21	2	1
P0CG50	UBC_MOUSE	Ubc	20	2	1
P10126	EF1A1_MOUSE	Eef1a1 Eef1a	2	1	1
P15864	H12_MOUSE	Hist1h1c H1f2	11	2	2
P16627	HS71L_MOUSE	Hspa1l Hsc70t	6	5	3
P16858	G3P_MOUSE	Gapdh Gapd	4	1	1
P18524	HVM53_MOUSE		9	3	1
P18526	HVM55_MOUSE		9	3	1
P20029	GRP78_MOUSE	Hspa5 Grp78	11	7	5
P43274	H14_MOUSE	Hist1h1e H1f4	11	2	2
P43277	H13_MOUSE	Hist1h1d H1f3	10	2	2
P52480	KPYM_MOUSE	Pkm Pk3 Pkm2 Pykm	13	6,5	5,5
P62631	EF1A2_MOUSE	Eef1a2 Eef1al Stn	2	1	1
P62830	RL23_MOUSE	Rpl23	14	1,5	1
P62983	RS27A_MOUSE	Rps27a Uba80 Ubcep1	10	2	1
P62984	RL40_MOUSE	Uba52 Ubcep2	12	2	1
P63017	HSP7C_MOUSE	Hspa8 Hsc70 Hsc73	27	30	12
Q01853	TERA_MOUSE	Vcp	4	2,5	2,5
Q07797	LG3BP_MOUSE	Lgals3bp Cycap Mama	8	5	3
Q3THE2	ML12B_MOUSE	Myl12b Mrlc2 Mylc2b	6	2	1
Q64467	G3PT_MOUSE	Gapdhs Gapd-s Gapds	3	1	1
Q6I6G8	HECW2_MOUSE	Hecw2 Kiaa1301 Nedl2	2	2	2
Q6IME9	K2C72_MOUSE	Krt72 Kb35 Krt72-ps	2	1	1
Q6URW6	MYH14_MOUSE	Myh14	1	2	2
Q8BMF4	ODP2_MOUSE	Dlat	3	2,5	1,5
Q8VDD5	MYH9_MOUSE	Myh9	11	21	14,5
Q99KV1	DJB11_MOUSE	Dnajb11	3	1	1
Q9JIY5	HTRA2_MOUSE	Htra2 Omi Prss25	5	2	2

GFP TAP-LC-MSMS in Ba/F3 rtTA3 cells, 2nd biological replicate

UniProtID	Entry_name	Gene_names	AvgSeqCov	AvgSpecCount	AvgPepCount
O35658	C1QBP_MOUSE	C1qbp Gc1qbp	5	1,5	1
O89023	TPP1_MOUSE	Tpp1 Cln2	6	2,5	2
P01868	IGHG1_MOUSE	Ighg1 Igh-4	11	8	3,5
P01869	IGH1M_MOUSE	Ighg1 Igh-4	9	8	3,5
P04104	K2C1_MOUSE	Krt1 Krt2-1	4	2	2
P05064	ALDOA_MOUSE	Aldoa Aldo1	6	2	2
P05213	TBA1B_MOUSE	Tuba1b Tuba2	14	6	4,5
P0CG49	UBB_MOUSE	Ubb	21	2	1
P0CG50	UBC_MOUSE	Ubc	20	2	1
P15864	H12_MOUSE	Hist1h1c H1f2	11	2,5	2
P16858	G3P_MOUSE	Gapdh Gapd	4	1	1
P18524	HVM53_MOUSE		9	2,5	1
P18526	HVM55_MOUSE		9	2,5	1
P20029	GRP78_MOUSE	Hspa5 Grp78	11	7	5
P43274	H14_MOUSE	Hist1h1e H1f4	11	2	2
P43276	H15_MOUSE	Hist1h1b H1f5	5	1	1
P43277	H13_MOUSE	Hist1h1d H1f3	10	2,5	2
P52480	KPYM_MOUSE	Pkm Pk3 Pkm2 Pykm	12	6	5
P60710	ACTB_MOUSE	Actb	8	2	2
P61514	RL37A_MOUSE	Rpl37a	37	2	2
P62806	H4_MOUSE	Hist1h4a; Hist1h4b H4-53; Hist1h4c H4-12; Hist1h4d; Hist1h4f; Hist1h4h; Hist1h4i; Hist1h4j; Hist1h4k; Hist1h4m; Hist2h4a Hist2h4; Hist4h4	23	3	2,5
P62983	RS27A_MOUSE	Rps27a Uba80 Ubcep1	10	2	1
P62984	RL40_MOUSE	Uba52 Ubcep2	12	2	1
P63017	HSP7C_MOUSE	Hspa8 Hsc70 Hsc73	29	29,5	14
P63260	ACTG_MOUSE	Actg1 Actg	8	2	2
P99027	RLA2_MOUSE	Rplp2	10	1	1
Q01853	TERA_MOUSE	Vcp	1	1	1
Q07797	LG3BP_MOUSE	Lgals3bp Cycap Mama	3	2	1
Q3THE2	ML12B_MOUSE	Myl12b Mrlc2 Mylc2b	6	2	1
Q60605	MYL6_MOUSE	Myl6 Myln	14	1,5	1,5
Q64467	G3PT_MOUSE	Gapdhs Gapd-s Gapds	3	1	1
Q6NXH9	K2C73_MOUSE	Krt73 Kb36	4	2	2
Q6URW6	MYH14_MOUSE	Myh14	1	2,5	2
Q6ZWV7	RL35_MOUSE	Rpl35	8	1	1
Q8BMF4	ODP2_MOUSE	Dlat	3	1,5	1
Q8CI43	MYL6B_MOUSE	Myl6b	6	1	1
Q8VDD5	MYH9_MOUSE	Myh9	11	19	14
Q99KV1	DJB11_MOUSE	Dnajb11	5	2	1,5
Q9CZX8	RS19_MOUSE	Rps19	6	1	1
Q9JIY5	HTRA2_MOUSE	Htra2 Omi Prss25	3	1	1

NRAS G12D TAP-LC-MSMS in Ba/F3 rtTA3 cells, 1st biological replicate

UniProtID	Entry_name	Gene_names	AvgSeqCov	AvgSpecCount	AvgPepCount
O89023	TPP1_MOUSE	Tpp1 Cln2	15	6,5	5
P01868	IGHG1_MOUSE	Ighg1 Igh-4	28	6	5,5
P01869	IGH1M_MOUSE	Ighg1 Igh-4	23	6	5,5
P05213	TBA1B_MOUSE	Tuba1b Tuba2	38	17,5	12
P08556	RASN_MOUSE	Nras	67	137,5	13
P0CG49	UBB_MOUSE	Ubb	21	3	1
P0CG50	UBC_MOUSE	Ubc	20	3	1
P10126	EF1A1_MOUSE	Eef1a1 Eef1a	8	4	3
P10518	HEM2_MOUSE	Alad Lv	5	1,5	1,5
P11499	HS90B_MOUSE	Hsp90ab1 Hsp84 Hsp84-1 Hspcb	4	2	2
P11983	TCPA_MOUSE	Tcp1 Cct1 Ccta	3	1,5	1,5
P15864	H12_MOUSE	Hist1h1c H1f2	16	4	4
P16858	G3P_MOUSE	Gapdh Gapd	14	5	3
P18524	HVM53_MOUSE		9	1	1
P18526	HVM55_MOUSE		9	1	1
P21107	TPM3_MOUSE	Tpm3 Tpm-5 Tpm5	9	3,333333333	3,333333333
P22752	H2A1_MOUSE	Hist1h2ab; Hist1h2ac; Hist1h2ad; Hist1h2ae; Hist1h2ag; Hist1h2ai; Hist1h2an; Hist1h2ao	22	2,5	2
P34884	MIF_MOUSE	Mif	8	1	1
P35700	PRDX1_MOUSE	Prdx1 Msp23 Paga Tdpx2	5	1	1
P43274	H14_MOUSE	Hist1h1e H1f4	15	3	3
P43277	H13_MOUSE	Hist1h1d H1f3	15	4	4
P48962	ADT1_MOUSE	Slc25a4 Anc1 Ant1	23	6	5
P50446	K2C6A_MOUSE	Krt6a Ker2 Krt2-6 Krt2-6a Krt6	4	5	2
P51881	ADT2_MOUSE	Slc25a5 Ant2	25	8	5,5
P52480	KPYM_MOUSE	Pkm Pk3 Pkm2 Pykm	13	6,666666667	5,333333333
P60710	ACTB_MOUSE	Actb	15	7	4
P61358	RL27_MOUSE	Rpl27	7	2	1
P61514	RL37A_MOUSE	Rpl37a	20	1	1
P62267	RS23_MOUSE	Rps23	8	1	1

P62751	RL23A_MOUSE	Rpl23a	8	1	1
P62806	H4_MOUSE	Hist1h4a; Hist1h4b H4-53; Hist1h4c H4-12; Hist1h4d; Hist1h4f; Hist1h4h; Hist1h4i; Hist1h4j; Hist1h4k; Hist1h4m; Hist2h4a Hist2h4; Hist4h4	12	1	1
P62830	RL23_MOUSE	Rpl23	14	1	1
P62852	RS25_MOUSE	Rps25	15	2	2
P62900	RL31_MOUSE	Rpl31	11	1	1
P62908	RS3_MOUSE	Rps3	11	2,5	2,5
P62983	RS27A_MOUSE	Rps27a Uba80 Ubcep1	10	3	1
P62984	RL40_MOUSE	Uba52 Ubcep2	12	3	1
P63017	HSP7C_MOUSE	Hspa8 Hsc70 Hsc73	27	19,5	13
P63260	ACTG_MOUSE	Actg1 Actg	15	7	4
P67984	RL22_MOUSE	Rpl22	9	1	1
P99024	TBB5_MOUSE	Tubb5	10	3,5	3,5
P99027	RLA2_MOUSE	Rplp2	18	1,5	1,5
Q01853	TERA_MOUSE	Vcp	6	3,5	3,5
Q03265	ATPA_MOUSE	Atp5a1	8	3	3
Q05920	PYC_MOUSE	Pc Pcx	2	2	2
Q07797	LG3BP_MOUSE	Lgals3bp Cycap Mama	10	4,5	3,5
Q3TCN2	PLBL2_MOUSE	Plbd2	2	1	1
Q3THE2	ML12B_MOUSE	Myl12b Mrlc2 Mylc2b	24	3,5	3
Q5SWU9	ACACA_MOUSE	Acaca Acac Gm738	2	5	5
Q60605	MYL6_MOUSE	Myl6 Myln	30	7	4,5
Q61414	K1C15_MOUSE	Krt15 Krt1-15	4	2	2
Q61781	K1C14_MOUSE	Krt14 Krt1-14	4	2	2
Q64523	H2A2C_MOUSE	Hist2h2ac Hist2h2ab	22	2,5	2
Q6GSS7	H2A2A_MOUSE	Hist2h2aa1; Hist2h2aa2	22	2,5	2
Q6IFX2	K1C42_MOUSE	Krt42 Ka22	4	2	2
Q6IME9	K2C72_MOUSE	Krt72 Kb35 Krt72-ps	2	3,5	1
Q6NXH9	K2C73_MOUSE	Krt73 Kb36	4	8	2
Q6URW6	MYH14_MOUSE	Myh14	1	1,5	1,5
Q791V5	MTCH2_MOUSE	Mtch2	14	2,5	2,5
Q8BFU2	H2A3_MOUSE	Hist3h2a	22	2,5	2
Q8BGZ7	K2C75_MOUSE	Krt75 Kb18	5	6	2,5
Q8BMF4	ODP2_MOUSE	Dlat	5	2,5	2
Q8CGP5	H2A1F_MOUSE	Hist1h2af	22	2,5	2
Q8CGP6	H2A1H_MOUSE	Hist1h2ah	22	2,5	2
Q8CGP7	H2A1K_MOUSE	Hist1h2ak	22	2,5	2
Q8R1M2	H2AJ_MOUSE	H2afj	22	2,5	2
Q8VBV7	CSN8_MOUSE	Cops8 Csn8	7	1	1
Q8VDD5	MYH9_MOUSE	Myh9	22	49	34
Q8VED5	K2C79_MOUSE	Krt79 Kb38	5	3	2
Q91ZA3	PCCA_MOUSE	Pcca	4	2	2
Q921Q7	RIN1_MOUSE	Rin1	15	9,5	7,5
Q922U2	K2C5_MOUSE	Krt5 Krt2-5	4	5	2
Q99KV1	DJB11_MOUSE	Dnajb11	8	2	2
Q99MN9	PCCB_MOUSE	Pccb	7	4	3,5
Q9CR62	M2OM_MOUSE	Slc25a11	6	1,5	1,5
Q9CXW4	RL11_MOUSE	Rpl11	8	1,5	1
Q9CZX8	RS19_MOUSE	Rps19	14	2	2
Q9D5Y0	CG031_MOUSE		4	2	2
Q9JHG7	PK3CG_MOUSE	Pik3cg Pi3kg1	4	4	3
Q9JIY5	HTRA2_MOUSE	Htra2 Omi Prss25	6	2,5	2,5
Q9JJI8	RL38_MOUSE	Rpl38	17	1	1
Q9QWL7	K1C17_MOUSE	Krt17 Krt1-17	5	2	2
Q9Z331	K2C6B_MOUSE	Krt6b K6-beta Krt2-6b	4	5	2

NRAS G12D TAP-LC-MSMS in Ba/F3 rtTA3 cells, 2nd biological replicate

UniProtID	Entry_name	Gene_names	AvgSeqCov	AvgSpecCount	AvgPepCount
O08638	MYH11_MOUSE	Myh11	3	8	6
O35658	C1QBP_MOUSE	C1qbp Gc1qbp	5	1,5	1
O54941	SMCE1_MOUSE	Smarce1 Baf57	3	1	1
O89023	TPP1_MOUSE	Tpp1 Cln2	15	7	5
P01837	IGKC_MOUSE		49	6,5	5
P01868	IGHG1_MOUSE	Ighg1 Igh-4	39	28	8
P01869	IGH1M_MOUSE	Ighg1 Igh-4	32	28	8
P04104	K2C1_MOUSE	Krt1 Krt2-1	5	6	4
P05064	ALDOA_MOUSE	Aldoa Aldo1	8	3,5	3
P05213	TBA1B_MOUSE	Tuba1b Tuba2	30	12,5	10
P08556	RASN_MOUSE	Nras	64	140	11
P08730	K1C13_MOUSE	Krt13 Krt1-13	8	4	4
P09528	FRIH_MOUSE	Fth1 Fth	12	2,5	2,5
P0CG49	UBB_MOUSE	Ubb	21	1	1
P0CG50	UBC_MOUSE	Ubc	20	1	1
P10126	EF1A1_MOUSE	Eef1a1 Eef1a	10	5	4
P10518	HEM2_MOUSE	Alad Lv	3	1	1
P11499	HS90B_MOUSE	Hsp90ab1 Hsp84 Hsp84-1 Hspcb	5	4	3,5
P11983	TCPA_MOUSE	Tcp1 Cct1 Ccta	4	2	2
P14115	RL27A_MOUSE	Rpl27a	22	3	3
P14131	RS16_MOUSE	Rps16	13	2	2
P15864	H12_MOUSE	Hist1h1c H1f2	16	5	4
P16858	G3P_MOUSE	Gapdh Gapd	11	3	2,5
P17809	GTR1_MOUSE	Slc2a1 Glut-1 Glut1	2	1	1
P18524	HVM53_MOUSE		9	1,5	1
P18526	HVM55_MOUSE		9	1,5	1
P20029	GRP78_MOUSE	Hspa5 Grp78	8	5,5	4
P21107	TPM3_MOUSE	Tpm3 Tpm-5 Tpm5	23	8,5	7,5
P22752	H2A1_MOUSE	Hist1h2ab; Hist1h2ac; Hist1h2ad; Hist1h2ae; Hist1h2ag; Hist1h2ai; Hist1h2an; Hist1h2ao	22	2,5	2
P29391	FRIL1_MOUSE	Ftl1 Ftl Ftl-1	21	2	2
P35700	PRDX1_MOUSE	Prdx1 Msp23 Paga Tdpx2	10	2	2
P35979	RL12_MOUSE	Rpl12	10	1	1
P38647	GRP75_MOUSE	Hspa9 Grp75 Hsp74 Hspa9a	4	2	2
P43274	H14_MOUSE	Hist1h1e H1f4	15	3	3
P43277	H13_MOUSE	Hist1h1d H1f3	15	5	4
P50446	K2C6A_MOUSE	Krt6a Ker2 Krt2-6 Krt2-6a Krt6	10	6,5	6
P51881	ADT2_MOUSE	Slc25a5 Ant2	25	7,5	6
P52480	KPYM_MOUSE	Pkm Pk3 Pkm2 Pykm	17	8,5	7
P60710	ACTB_MOUSE	Actb	38	15	11
P61358	RL27_MOUSE	Rpl27	28	3	3
P61514	RL37A_MOUSE	Rpl37a	20	1	1
P61982	1433G_MOUSE	Ywhag	7	1,5	1,5
P62267	RS23_MOUSE	Rps23	8	1	1
P62270	RS18_MOUSE	Rps18	14	3	3
P62274	RS29_MOUSE	Rps29	27	2	1,5
P62281	RS11_MOUSE	Rps11	12	2	2
P62301	RS13_MOUSE	Rps13	8	1	1
P62702	RS4X_MOUSE	Rps4x Rps4	16	4	4
P62751	RL23A_MOUSE	Rpl23a	28	6	6
P62806	H4_MOUSE	Hist1h4a; Hist1h4b H4-53; Hist1h4c H4-12; Hist1h4d; Hist1h4f; Hist1h4h; Hist1h4i; Hist1h4j; Hist1h4k; Hist1h4m; Hist2h4a Hist2h4; Hist4h4	34	4,5	3,5
P62830	RL23_MOUSE	Rpl23	23	2,5	2,5
P62852	RS25_MOUSE	Rps25	19	2,5	2,5
P62900	RL31_MOUSE	Rpl31	18	2,5	2
P62908	RS3_MOUSE	Rps3	29	7	6,5
P62960	YBOX1_MOUSE	Ybx1 Msy-1 Msy1 Nsep1 Yb1	5	1	1
P62983	RS27A_MOUSE	Rps27a Uba80 Ubcep1	10	1	1
P62984	RL40_MOUSE	Uba52 Ubcep2	12	1	1
P63017	HSP7C_MOUSE	Hspa8 Hsc70 Hsc73	34	23,5	17
P63260	ACTG_MOUSE	Actg1 Actg	35	15	10,5
P63276	RS17_MOUSE	Rps17	9	1,5	1,5
P67984	RL22_MOUSE	Rpl22	12	1	1
P68033	ACTC_MOUSE	Actc1 Actc	25	10	8
P68040	GBLP_MOUSE	Gnb2l1 Gnb2-rs1	6	2	2
P68134	ACTS_MOUSE	Acta1 Acta	25	10	8
P68368	TBA4A_MOUSE	Tuba4a Tuba4	26	10,5	8,5
P68372	TBB4B_MOUSE	Tubb4b Tubb2c	25	11	9
P80318	TCPG_MOUSE	Cct3 Cctg	2	1	1
P99024	TBB5_MOUSE	Tubb5	23	10,5	8,5

P99027	RLA2_MOUSE	Rplp2	55	5	4,5
Q01768	NDKB_MOUSE	Nme2	20	3,5	3
Q01853	TERA_MOUSE	Vcp	9	7	5,5
Q03265	ATPA_MOUSE	Atp5a1	7	3	3
Q05920	PYC_MOUSE	Pc Pcx	5	5	4,5
Q07797	LG3BP_MOUSE	Lgals3bp Cycap Mama	11	4	4
Q3TCN2	PLBL2_MOUSE	Plbd2	2	1	1
Q3THE2	ML12B_MOUSE	Myl12b Mrlc2 Mylc2b	27	4	3,5
Q497I4	KRT35_MOUSE	Krt35 Krt1-24	7	2	2
Q5SWU9	ACACA_MOUSE	Acaca Acac Gm738	7	13,5	13,5
Q60605	MYL6_MOUSE	Myl6 Myln	30	8,5	5
Q61753	SERA_MOUSE	Phgdh	7	3	3
Q64523	H2A2C_MOUSE	Hist2h2ac Hist2h2ab	22	2,5	2
Q6GSS7	H2A2A_MOUSE	Hist2h2aa1; Hist2h2aa2	22	2,5	2
Q6IME9	K2C72_MOUSE	Krt72 Kb35 Krt72-ps	4	2,5	2
Q6NXH9	K2C73_MOUSE	Krt73 Kb36	6	5,5	3,5
Q6URW6	MYH14_MOUSE	Myh14	2	5,5	4,5
Q6ZWU9	RS27_MOUSE	Rps27	28	2	2
Q6ZWV7	RL35_MOUSE	Rpl35	15	2	2
Q6ZWY3	RS27L_MOUSE	Rps27l	28	2	2
Q78IK2	USMG5_MOUSE	Usmg5 Dapit	26	1	1
Q791V5	MTCH2_MOUSE	Mtch2	17	4	3
Q8BFU2	H2A3_MOUSE	Hist3h2a	22	2,5	2
Q8BGZ7	K2C75_MOUSE	Krt75 Kb18	8	4	4
Q8BH59	CMC1_MOUSE	Slc25a12 Aralar1	4	2	2
Q8BMF4	ODP2_MOUSE	Dlat	6	3,5	2,5
Q8CGP5	H2A1F_MOUSE	Hist1h2af	22	2,5	2
Q8CGP6	H2A1H_MOUSE	Hist1h2ah	22	2,5	2
Q8CGP7	H2A1K_MOUSE	Hist1h2ak	22	2,5	2
Q8R1M2	H2AJ_MOUSE	H2afj	22	2,5	2
Q8VBV7	CSN8_MOUSE	Cops8 Csn8	7	1	1
Q8VDD5	MYH9_MOUSE	Myh9	33	85,5	55
Q8VED5	K2C79_MOUSE	Krt79 Kb38	6	3	3
Q8VEK3	HNRPU_MOUSE	Hnrnpu Hnrpu	2	1	1
Q91VR2	ATPG_MOUSE	Atp5c1	4	1,5	1
Q91ZA3	PCCA_MOUSE	Pcca	8	7	5
Q921Q7	RIN1_MOUSE	Rin1	18	11	10
Q922B2	SYDC_MOUSE	Dars	5	2	2
Q99JR1	SFXN1_MOUSE	Sfxn1 F	4	1	1
Q99KP6	PRP19_MOUSE	Prpf19 Prp19 Snev	6	2	2
Q99KQ4	NAMPT_MOUSE	Nampt Pbef1	3	1	1
Q99KV1	DJB11_MOUSE	Dnajb11	12	3	3
Q99MN1	SYK_MOUSE	Kars	3	1,5	1,5
Q99MN9	PCCB_MOUSE	Pccb	13	6	5,5
Q99N57	RAF1_MOUSE	Raf1 Craf	9	4,5	4,5
Q9CR62	M2OM_MOUSE	Slc25a11	6	1,5	1,5
Q9CXW4	RL11_MOUSE	Rpl11	8	1	1
Q9CZX8	RS19_MOUSE	Rps19	21	3,5	3,5
Q9DCH4	EIF3F_MOUSE	Eif3f Eif3s5	8	2	2
Q9DCT5	SDF2_MOUSE	Sdf2	16	2	1,5
Q9ESP1	SDF2L_MOUSE	Sdf2l1	17	3	2
Q9JHG7	PK3CG_MOUSE	Pik3cg Pi3kg1	6	5,5	5,5
Q9JIY5	HTRA2_MOUSE	Htra2 Omi Prss25	5	2	2
Q9JJZ2	TBA8_MOUSE	Tuba8	22	10	7
Q9JKB3	YBOX3_MOUSE	Ybx3 Csda Msy4	5	1	1
Q9QZD9	EIF3I_MOUSE	Eif3i Eif3s2 Trip1	8	2,5	2
Q9Z2C8	YBOX2_MOUSE	Ybx2 Msy2	5	1	1

Supplementary Table 4
Protein identification of GFP and MLKL S358D TAP-LC-MSMS in HT-29 RIEP cells

Column	Description
UniProtID	UniprotKB ID
Entry_name	UniprotKB entry name
Gene_names	UniprotKB entry derived gene names
AvgSeqCov	Average sequence coverage in two technical replicates
AvgSpecCount	Average spectral counts in two technical replicates
AvgPepCount	Average peptide counts in two technical replicates

GFP TAP-LC-MSMS in HT-29 RIEP cells, 1st biological replicate

UniProtID	Entry_name	Gene_names	AvgSeqCov	AvgSpecCount	AvgPepCount
A5A3E0	POTEF_HUMAN	POTEF A26C1B	1	1	1
O14773	TPP1_HUMAN	TPP1 CLN2 GIG1 UNQ267/PRO304	3	1	1
O43464	HTRA2_HUMAN	HTRA2 OMI PRSS25	6	2	2
P02533	K1C14_HUMAN	KRT14	6	3	3
P02538	K2C6A_HUMAN	KRT6A K6A KRT6D	7	4	4
P04259	K2C6B_HUMAN	KRT6B K6B KRTL1	7	4	4
P04264	K2C1_HUMAN	KRT1 KRTA	15	9	8,5
P05387	RLA2_HUMAN	RPLP2 D11S2243E RPP2	17	1	1
P05783	K1C18_HUMAN	KRT18 CYK18 PIG46	4	2	2
P05787	K2C8_HUMAN	KRT8 CYK8	7	4	3,5
P07437	TBB5_HUMAN	TUBB TUBB5 OK/SW-cl.56	18	7	6,5
P08107	NA	NA	24	34,5	11,5
P0CG47	UBB_HUMAN	UBB	38	14	2
P0CG48	UBC_HUMAN	UBC	38	14	2
P11021	GRP78_HUMAN	HSPA5 GRP78	21	26	9
P11142	HSP7C_HUMAN	HSPA8 HSC70 HSP73 HSPA10	47	66,5	26
P13645	K1C10_HUMAN	KRT10 KPP	29	22	14,5
P17066	HSP76_HUMAN	HSPA6 HSP70B'	13	30,5	8
P19013	K2C4_HUMAN	KRT4 CYK4	4	2	2
P34931	HS71L_HUMAN	HSPA1L	12	26,5	7,5
P35908	K22E_HUMAN	KRT2 KRT2A KRT2E	16	9,5	9,5
P38646	GRP75_HUMAN	HSPA9 GRP75 HSPA9B mt-HSP70	17	8,5	8,5
P48668	K2C6C_HUMAN	KRT6C KRT6E	7	4	4
P60709	ACTB_HUMAN	ACTB	5	1,5	1,5
P62736	ACTA_HUMAN	ACTA2 ACTSA ACTVS GIG46	5	1,5	1,5
P62829	RL23_HUMAN	RPL23	14	8	1
P62979	RS27A_HUMAN	RPS27A UBA80 UBCEP1	19	14	2
P62987	RL40_HUMAN	UBA52 UBCEP2	23	14	2
P63261	ACTG_HUMAN	ACTG1 ACTG	5	1,5	1,5
P63267	ACTH_HUMAN	ACTG2 ACTA3 ACTL3 ACTSG	5	1,5	1,5
P68032	ACTC_HUMAN	ACTC1 ACTC	5	1,5	1,5
P68104	EF1A1_HUMAN	EEF1A1 EEF1A EF1A LENG7	2	1	1
P68133	ACTS_HUMAN	ACTA1 ACTA	5	1,5	1,5
P68363	TBA1B_HUMAN	TUBA1B	33	14,5	10
P68366	TBA4A_HUMAN	TUBA4A TUBA1	27	10	7,5
P68371	TBB4B_HUMAN	TUBB4B TUBB2C	10	5	4
Q05639	EF1A2_HUMAN	EEF1A2 EEF1AL STN	2	1	1
Q08380	LG3BP_HUMAN	LGALS3BP M2BP	5	2	2
Q562R1	ACTBL_HUMAN	ACTBL2	5	1,5	1,5
Q5VTE0	EF1A3_HUMAN	EEF1A1P5 EEF1AL3	2	1	1
Q6S8J3	POTEE_HUMAN	POTEE A26C1A POTE2	1	1	1
Q7Z5K2	WAPL_HUMAN	WAPAL FOE KIAA0261 WAPL	3	2	2
Q8IUS5	EPHX4_HUMAN	EPHX4 ABHD7 EPHXRP	12	2	2
Q8NB16	MLKL_HUMAN	MLKL	4	2	2
Q8WZ42	TITIN_HUMAN	TTN	0	8	3
Q9BQE3	TBA1C_HUMAN	TUBA1C TUBA6	33	14,5	10
Q9BVA1	TBB2B_HUMAN	TUBB2B	11	4	4
Q9BYX7	ACTBM_HUMAN	POTEKP ACTBL3 FKSG30	4	1	1
Q9UBS4	DJB11_HUMAN	DNAJB11 EDJ ERJ3 HDJ9 PSEC0121 UNQ537/PRO1080	8	2,5	2
Q9Y572	RIPK3_HUMAN	RIPK3 RIP3	8	4,5	2

GFP TAP-LC-MSMS in HT-29 RIEP cells, 2nd biological replicate

UniProtID	Entry_name	Gene_names	AvgSeqCov	AvgSpecCount	AvgPepCount
O14773	TPP1_HUMAN	TPP1 CLN2 GIG1 UNQ267/PRO304	3	1,5	1
O43464	HTRA2_HUMAN	HTRA2 OMI PRSS25	6	2	2
O60271	JIP4_HUMAN	SPAG9 HSS KIAA0516 MAPK8IP4 SYD1 HLC6	1	1	1
O95678	K2C75_HUMAN	KRT75 K6HF KB18	2	1	1
O95816	BAG2_HUMAN	BAG2	5	1	1
P02538	K2C6A_HUMAN	KRT6A K6A KRT6D	2	1	1
P04259	K2C6B_HUMAN	KRT6B K6B KRTL1	2	1	1
P04264	K2C1_HUMAN	KRT1 KRTA	8	4	4
P04792	HSPB1_HUMAN	HSPB1 HSP27 HSP28	5	1	1
P05141	ADT2_HUMAN	SLC25A5 ANT2	7	2	2

P05387	RLA2_HUMAN	RPLP2 D11S2243E RPP2	23	1,5	1,5
P05787	K2C8_HUMAN	KRT8 CYK8	5	2,5	2,5
P07437	TBB5_HUMAN	TUBB TUBB5 OK/SW-cl.56	27	14	10
P08107	NA	NA	31	41,33333333	14
P08729	K2C7_HUMAN	KRT7 SCL	4	2	2
P09972	ALDOC_HUMAN	ALDOC ALDC	8	2	2
P0CG47	UBB_HUMAN	UBB	50	16	3
P0CG48	UBC_HUMAN	UBC	50	16	3
P11021	GRP78_HUMAN	HSPA5 GRP78	21	25	10
P11142	HSP7C_HUMAN	HSPA8 HSC70 HSP73 HSPA10	49	80,5	28
P12235	ADT1_HUMAN	SLC25A4 ANT1	7	2	2
P12236	ADT3_HUMAN	SLC25A6 ANT3 CDABP0051	7	2	2
P13645	K1C10_HUMAN	KRT10 KPP	7	4,5	3,5
P13647	K2C5_HUMAN	KRT5	2	1	1
P17066	HSP76_HUMAN	HSPA6 HSP70B'	13	32	7,5
P24534	EF1B_HUMAN	EEF1B2 EEF1B EF1B	7	1	1
P34931	HS71L_HUMAN	HSPA1L	17	26	8,5
P35527	K1C9_HUMAN	KRT9	3	2	2
P35908	K22E_HUMAN	KRT2 KRT2A KRT2E	2	1	1
P38646	GRP75_HUMAN	HSPA9 GRP75 HSPA9B mt-HSP70	18	10	9
P48668	K2C6C_HUMAN	KRT6C KRT6E	2	1	1
P55072	TERA_HUMAN	VCP	4	2	2
P60709	ACTB_HUMAN	ACTB	11	3	3
P61981	1433G_HUMAN	YWHAG	6	1	1
P62829	RL23_HUMAN	RPL23	17	7,5	1,5
P62979	RS27A_HUMAN	RPS27A UBA80 UBCEP1	24	16	3
P62987	RL40_HUMAN	UBA52 UBCEP2	30	16	3
P63261	ACTG_HUMAN	ACTG1 ACTG	11	3	3
P68104	EF1A1_HUMAN	EEF1A1 EEF1A EF1A LENG7	3	1,5	1,5
P68363	TBA1B_HUMAN	TUBA1B	33	20,5	10
P68366	TBA4A_HUMAN	TUBA4A TUBA1	25	15,5	7
P68371	TBB4B_HUMAN	TUBB4B TUBB2C	20	11	8
Q05639	EF1A2_HUMAN	EEF1A2 EEF1AL STN	3	1,5	1,5
Q5VTE0	EF1A3_HUMAN	EEF1A1P5 EEF1AL3	3	1,5	1,5
Q5XKE5	K2C79_HUMAN	KRT79 K6L KB38 KRT6L	2	1	1
Q86X55	CARM1_HUMAN	CARM1 PRMT4	4	2	2
Q9BQE3	TBA1C_HUMAN	TUBA1C TUBA6	33	19,5	10
Q9BVA1	TBB2B_HUMAN	TUBB2B	19	10	7
Q9UBS4	DJB11_HUMAN	DNAJB11 EDJ ERJ3 HDJ9 PSEC0121 UNQ537/PRO1080	8	2	2
Q9UHV9	PFD2_HUMAN	PFDN2 PFD2 HSPC231	9	1	1

MLKL S358D TAP-LC-MSMS in HT-29 RIEP cells, 1st biological replicate

UniProtID	Entry_name	Gene_names	AvgSeqCov	AvgSpecCount	AvgPepCount
O00294	TULP1_HUMAN	TULP1 TUBL1	5	2	2
O14773	TPP1_HUMAN	TPP1 CLN2 GIG1 UNQ267/PRO304	3	1	1
O43464	HTRA2_HUMAN	HTRA2 OMI PRSS25	3	1	1
O60884	DNJA2_HUMAN	DNAJA2 CPR3 HIRIP4	4	1	1
P02533	K1C14_HUMAN	KRT14	13	5,5	5
P02538	K2C6A_HUMAN	KRT6A K6A KRT6D	7	4	3
P04259	K2C6B_HUMAN	KRT6B K6B KRTL1	5	3	2
P04264	K2C1_HUMAN	KRT1 KRTA	14	7,5	7
P05141	ADT2_HUMAN	SLC25A5 ANT2	4	1	1
P05161	ISG15_HUMAN	ISG15 G1P2 UCRP	12	1	1
P05387	RLA2_HUMAN	RPLP2 D11S2243E RPP2	38	2,5	2
P05787	K2C8_HUMAN	KRT8 CYK8	4	2	2
P07355	ANXA2_HUMAN	ANXA2 ANX2 ANX2L4 CAL1H LPC2D	6	1,5	1,5
P07437	TBB5_HUMAN	TUBB TUBB5 OK/SW-cl.56	31	14,5	9,5
P07900	HS90A_HUMAN	HSP90AA1 HSP90A HSPC1 HSPCA	20	15,5	15
P08107	NA	NA	30	29,5	12,5
P08238	HS90B_HUMAN	HSP90AB1 HSP90B HSPC2 HSPCB	28	23,5	19,5
P08708	RS17_HUMAN	RPS17 RPS17L	25	2	2
P08779	K1C16_HUMAN	KRT16 KRT16A	9	4,5	4
P0CG47	UBB_HUMAN	UBB	21	4,5	1
P0CG48	UBC_HUMAN	UBC	21	4,5	1
P0CW22	RS17_HUMAN	RPS17 RPS17L	25	2	2
P11021	GRP78_HUMAN	HSPA5 GRP78	9	18,5	4
P11142	HSP7C_HUMAN	HSPA8 HSC70 HSP73 HSPA10	45	57,5	23
P12235	ADT1_HUMAN	SLC25A4 ANT1	4	1	1
P12236	ADT3_HUMAN	SLC25A6 ANT3 CDABP0051	4	1	1
P13645	K1C10_HUMAN	KRT10 KPP	18	13,5	8
P13647	K2C5_HUMAN	KRT5	2	1	1
P17066	HSP76_HUMAN	HSPA6 HSP70B'	13	24	7
P19013	K2C4_HUMAN	KRT4 CYK4	4	2,5	2
P23396	RS3_HUMAN	RPS3 OK/SW-cl.26	5	1	1
P30048	PRDX3_HUMAN	PRDX3 AOP1	10	2	2
P34931	HS71L_HUMAN	HSPA1L	15	23	7
P35030	TRY3_HUMAN	PRSS3 PRSS4 TRY3 TRY4	4	1	1

P35232	PHB_HUMAN	PHB	7	1	1
P35527	K1C9_HUMAN	KRT9	11	4,5	4,5
P35908	K22E_HUMAN	KRT2 KRT2A KRT2E	4	3	2,5
P38646	GRP75_HUMAN	HSPA9 GRP75 HSPA9B mt-HSP70	9	4,5	4,5
P46782	RS5_HUMAN	RPS5	10	1	1
P48668	K2C6C_HUMAN	KRT6C KRT6E	7	4	3
P51784	UBP11_HUMAN	USP11 UHX1	1	1	1
P52597	HNRPF_HUMAN	HNRNPF HNRPF	6	1,5	1,5
P54652	HSP72_HUMAN	HSPA2	14	38	9
P55072	TERA_HUMAN	VCP	6	3	3
P61247	RS3A_HUMAN	RPS3A FTE1 MFTL	6	1	1
P61513	RL37A_HUMAN	RPL37A	20	1	1
P61978	HNRPK_HUMAN	HNRNPK HNRPK	3	1	1
P62249	RS16_HUMAN	RPS16	7	1	1
P62280	RS11_HUMAN	RPS11	16	2	2
P62829	RL23_HUMAN	RPL23	14	7,5	1
P62854	RS26_HUMAN	RPS26	13	1	1
P62913	RL11_HUMAN	RPL11	8	1	1
P62979	RS27A_HUMAN	RPS27A UBA80 UBCEP1	10	4,5	1
P62987	RL40_HUMAN	UBA52 UBCEP2	12	4,5	1
P63208	SKP1_HUMAN	SKP1 EMC19 OCP2 SKP1A TCEB1L	7	1	1
P68104	EF1A1_HUMAN	EEF1A1 EEF1A EF1A LENG7	4	3,5	2
P68363	TBA1B_HUMAN	TUBA1B	34	13	10
P68366	TBA4A_HUMAN	TUBA4A TUBA1	25	8,5	7
P68371	TBB4B_HUMAN	TUBB4B TUBB2C	26	11,5	8,5
Q05639	EF1A2_HUMAN	EEF1A2 EEF1AL STN	4	3,5	2
Q06830	PRDX1_HUMAN	PRDX1 PAGA PAGB TDPX2	5	1	1
Q08380	LG3BP_HUMAN	LGALS3BP M2BP	7	2,5	2,5
Q12931	TRAP1_HUMAN	TRAP1 HSP75	2	1	1
Q13085	ACACA_HUMAN	ACACA ACAC ACC1 ACCA	4	7	6,333333333
Q14257	RCN2_HUMAN	RCN2 ERC55	8	1,5	1,5
Q16543	CDC37_HUMAN	CDC37 CDC37A	16	5	4
Q58FF8	H90B2_HUMAN	HSP90AB2P HSP90BB	12	4	4
Q5VTE0	EF1A3_HUMAN	EEF1A1P5 EEF1AL3	4	3,5	2
Q8NB16	MLKL_HUMAN	MLKL	84	609	54,5
Q8NCM8	DYHC2_HUMAN	DYNC2H1 DHC1B DHC2 DNCH2 DYH1B KIAA1997	1	3	2
Q92598	HS105_HUMAN	HSPH1 HSP105 HSP110 KIAA0201	7	4	4
Q9H0C2	ADT4_HUMAN	SLC25A31 AAC4 ANT4 SFEC	4	1	1
Q9H254	SPTN4_HUMAN	SPTBN4 KIAA1642 SPTBN3	1	4	3
Q9UBS4	DJB11_HUMAN	DNAJB11 EDJ ERJ3 HDJ9 PSEC0121 UNQ537/PRO1080	8	4,5	2
Q9Y572	RIPK3_HUMAN	RIPK3 RIP3	38	19,5	12,5

MLKL S358D TAP-LC-MSMS in HT-29 RIEP cells, 2nd biological replicate

UniProtID	Entry_name	Gene_names	AvgSeqCov	AvgSpecCount	AvgPepCount
O14773	TPP1_HUMAN	TPP1 CLN2 GIG1 UNQ267/PRO304	3	1	1
O43390	HNRPR_HUMAN	HNRNPR HNRPR	2	1	1
P07437	TBB5_HUMAN	TUBB TUBB5 OK/SW-cl.56	13	8	4,5
P07900	HS90A_HUMAN	HSP90AA1 HSP90A HSPC1 HSPCA	12	10,5	9,5
P08107	NA	NA	19	28,5	7,5
P08238	HS90B_HUMAN	HSP90AB1 HSP90B HSPC2 HSPCB	16	14	11,5
P0CG47	UBB_HUMAN	UBB	21	6,5	1
P0CG48	UBC_HUMAN	UBC	21	6,5	1
P11021	GRP78_HUMAN	HSPA5 GRP78	8	17	4
P11142	HSP7C_HUMAN	HSPA8 HSC70 HSP73 HSPA10	36	53,5	18
P14618	KPYM_HUMAN	PKM OIP3 PK2 PK3 PKM2	2	1,5	1,5
P17066	HSP76_HUMAN	HSPA6 HSP70B'	10	26	5,5
P30048	PRDX3_HUMAN	PRDX3 AOP1	5	1	1
P34931	HS71L_HUMAN	HSPA1L	10	19,5	4,5
P35030	TRY3_HUMAN	PRSS3 PRSS4 TRY3 TRY4	4	1	1
P38646	GRP75_HUMAN	HSPA9 GRP75 HSPA9B mt-HSP70	8	4	4
P46782	RS5_HUMAN	RPS5	10	1	1
P52597	HNRPF_HUMAN	HNRNPF HNRPF	6	1,5	1,5
P54652	HSP72_HUMAN	HSPA2	13	33	8
P55072	TERA_HUMAN	VCP	3	1,5	1,5
P61247	RS3A_HUMAN	RPS3A FTE1 MFTL	6	1	1
P61978	HNRPK_HUMAN	HNRNPK HNRPK	3	1	1
P62829	RL23_HUMAN	RPL23	14	11	1
P62913	RL11_HUMAN	RPL11	8	1	1
P62979	RS27A_HUMAN	RPS27A UBA80 UBCEP1	10	6,5	1
P62987	RL40_HUMAN	UBA52 UBCEP2	12	6,5	1
P68363	TBA1B_HUMAN	TUBA1B	32	17	9,5
P68366	TBA4A_HUMAN	TUBA4A TUBA1	22	8	6
P68371	TBB4B_HUMAN	TUBB4B TUBB2C	8	4,5	3
Q06830	PRDX1_HUMAN	PRDX1 PAGA PAGB TDPX2	5	1	1
Q08380	LG3BP_HUMAN	LGALS3BP M2BP	6	2	2
Q12931	TRAP1_HUMAN	TRAP1 HSP75	2	1	1
Q13085	ACACA_HUMAN	ACACA ACAC ACC1 ACCA	1	2,5	2,5

Q14257	RCN2_HUMAN	RCN2 ERC55	8	1,5	1,5
Q16543	CDC37_HUMAN	CDC37 CDC37A	11	5,5	2,5
Q5M9N0	CD158_HUMAN	CCDC158	3	2	2
Q8NB16	MLKL_HUMAN	MLKL	71	537	47
Q92598	HS105_HUMAN	HSPH1 HSP105 HSP110 KIAA0201	6	4	4
Q9UBS4	DJB11_HUMAN	DNAJB11 EDJ ERJ3 HDJ9 PSEC0121 UNQ537/PRO1080	8	4	2
Q9Y572	RIPK3_HUMAN	RIPK3 RIP3	20	14	7
